# Low-carbohydrate diets containing plant-derived fat but not animal-derived fat ameliorate heart failure

**DOI:** 10.1038/s41598-023-30821-7

**Published:** 2023-03-09

**Authors:** Satoshi Bujo, Haruhiro Toko, Kaoru Ito, Satoshi Koyama, Masato Ishizuka, Masahiko Umei, Haruka Yanagisawa-Murakami, Jiaxi Guo, Bowen Zhai, Chunxia Zhao, Risa Kishikawa, Norifumi Takeda, Kensuke Tsushima, Yuichi Ikeda, Eiki Takimoto, Hiroyuki Morita, Mutsuo Harada, Issei Komuro

**Affiliations:** 1 Department of Cardiovascular Medicine, Graduate School of Medicine, The University of Tokyo, 7-3-1 Hongo, Bunkyo-ku, Tokyo, 113-8655 Japan; 2 Department of Advanced Translational Research and Medicine in Management of Pulmonary Hypertension, Graduate School of Medicine, The University of Tokyo, Bunkyo-ku, Tokyo, 113-8655 Japan; 3 Laboratory for Cardiovascular Genomics and Informatics, RIKEN Center for Integrative Medical Sciences, Tsurumi-ku, Yokohama, Kanagawa 230-0045 Japan; 4 Department of Advanced Clinical Science and Therapeutics, Graduate School of Medicine, The University of Tokyo, Bunkyo-ku, Tokyo, 113-8655 Japan

**Keywords:** Heart failure, Fatty acids

## Abstract

Cardiovascular disease (CVD) is a global health burden in the world. Although low-carbohydrate diets (LCDs) have beneficial effects on CVD risk, their preventive effects remain elusive. We investigated whether LCDs ameliorate heart failure (HF) using a murine model of pressure overload. LCD with plant-derived fat (LCD-P) ameliorated HF progression, whereas LCD with animal-derived fat (LCD-A) aggravated inflammation and cardiac dysfunction. In the hearts of LCD-P-fed mice but not LCD-A, fatty acid oxidation-related genes were highly expressed, and peroxisome proliferator-activated receptor α (PPARα), which regulates lipid metabolism and inflammation, was activated. Loss- and gain-of-function experiments indicated the critical roles of PPARα in preventing HF progression. Stearic acid, which was more abundant in the serum and heart of LCD-P-fed mice, activated PPARα in cultured cardiomyocytes. We highlight the importance of fat sources substituted for reduced carbohydrates in LCDs and suggest that the LCD-P-stearic acid-PPARα pathway as a therapeutic target for HF.

## Introduction

Cardiovascular disease (CVD) is a global health burden with increasing prevalence and mortality rates^1^. CVDs result from unhealthy lifestyles, as well as genetic factors, and some risk factors, such as smoking status, physical inactivity, obesity, poor dietary habits, high blood glucose levels, high serum cholesterol levels, and high blood pressure, can be changed through lifestyle modifications^2,3^. Dietary intervention is one of the most effective methods of reducing these risk factors. Low-carbohydrate diets (LCDs) have been used for weight loss^4^, and many randomized clinical trials show that LCDs induce beneficial changes in CVD risk factors, such as hypertension, dyslipidemia, and diabetes^5–8^. However, it has remained unclear whether LCDs reduce the incidence of CVDs and related death^9–11^. Recent clinical studies have suggested the importance of macronutrients substituted for reduced carbohydrates in LCDs^12–14^. LCDs with animal-derived fat and protein sources were associated with higher CVD mortality, whereas LCDs with plant-derived fat and protein sources were associated with lower CVD mortality. Despite these distinct effects of LCDs on CVDs, their pathological mechanisms have not yet been elucidated.

Peroxisome proliferator-activated receptor α (PPARα) is a transcription factor that regulates lipid metabolism genes by sensing fatty acids. PPARα promotes fatty acid oxidation (FAO) in the muscle, liver, and adipose tissue, increasing energy expenditure and reducing body fat. PPARα agonists have been clinically used to treat dyslipidemia. In addition to these effects of lowering the CVD risk factors, anti-inflammatory effects are also reported in liver and blood vessels^15^. Since heart failure (HF), a leading cause of CVD-related death, is characterized by low FAO rates with energy metabolism disruption, PPARα activation is expected to rescue the failing heart^16^. There were several studies concerning the effects of LCDs on HF. A lard diet significantly hastened death as compared with a high-carbohydrate diet, whereas a linoleate diet significantly delayed the death of spontaneously hypertensive HF rats^17^. Additionally, strict restriction of carbohydrates attenuated the progression of pathological hypertrophy and systolic dysfunction in a pressure-overload model, and LCDs with high-protein supplementation and high-fat supplementation showed cardioprotection through distinct mechanisms^18^. These findings suggest that the beneficial effects of LCDs on HF depend on the substitute supplementation in LCDs.

In this study, we investigated whether the effects of LCDs on HF differed depending on the fat sources of substitute supplementation in LCDs and explored the molecular mechanisms of these distinct effects.

## Results

### Differences in the effects of LCDs on cardiac function depending on the fat source

**Figure 1:**
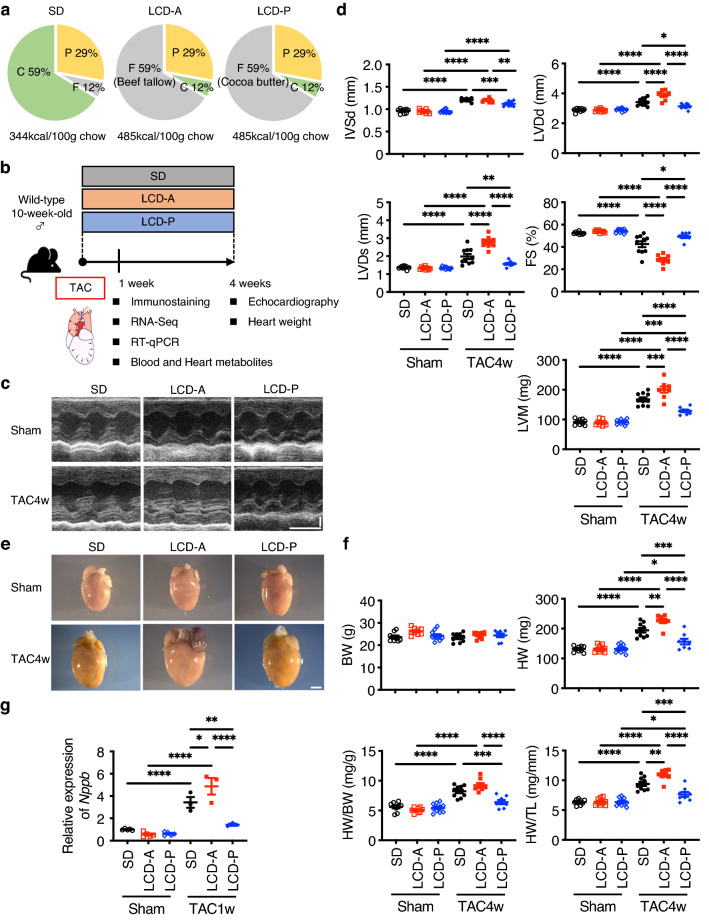
Distinct effects of LCDs on cardiac hypertrophy by fat sources. **(a)** Pie chart showing the proportions of calories from carbohydrate (C), fat (F), and protein (P) in each diet. SD, standard diet; LCD-A, low-carbohydrate diet with animal-derived fat; LCD-P, low-carbohydrate diet with plant-derived fat. (**b)** Experimental outline. Wild-type mice were fed with the indicated diets for 4 weeks starting from the day of transverse aortic constriction (TAC) or sham surgery. Analysis was performed at 1- or 4-weeks post-surgery. (**c)** Representative images of motion-mode (M-mode) echocardiography taken at 4-weeks post-surgery. Vertical scale bar, 1 mm. Transverse scale bar, 100 ms. (**d)** Cardiac function was assessed by M-mode echocardiography at 4-weeks post-TAC or post-sham surgery (*n* = 8–10). *IVSd* diastolic interventricular-septum thickness, *LVDd* left ventricular end-diastolic dimension, *LVDs* left ventricular end-systolic dimension, *LVM* left ventricular mass, *FS* fractional shortening. (**e**) Representative gross anatomies of the heart at 4-weeks post-TAC or post-sham surgery. Scale bar, 2 mm. (**f**) Body weight (BW), heart weight (HW), HW:BW ratio, and HW:tibial length (TL) ratio at 4-weeks post-TAC or post-sham surgery (*n* = 8–10). (**g)** mRNA level of *Nppb* in the heart at 1-week post-TAC or post-sham surgery (*n* = 3–5). Data represent the mean ± SEM. Statistical significance was analyzed by two-way ANOVA, followed by Holm–Sidak’s post-hoc test. *P < 0.05, **P < 0.01, ***P < 0.001, ****P < 0.0001.

To examine whether the effects of LCDs on cardiac function depend on the fat source in LCDs, we prepared two types of LCDs (12% carbohydrate and 59% fat of total energy) as follows: LCD with animal-derived fat (beef tallow; LCD-A) and LCD with plant-derived fat (cocoa butter; LCD-P). A high-carbohydrate standard diet (59% carbohydrate and 12% fat of total energy; SD) was used as a control (Fig. 1a). There was no significant difference in calorie intake and serum triglyceride levels between mice fed these two types of LCDs (Supplementary Fig. 1a,b). We provided mice with SD, LCD-A, or LCD-P for 4 weeks starting from the day of pressure-overload or sham surgery (Fig. 1b). Pressure overload induced by transverse aortic constriction (TAC) increased the wall thickness and dimensions of the left ventricle (LV) and reduced cardiac systolic function in mice on SD (Fig. 1c,d). LCD-P attenuated these morphologic changes, including LV hypertrophy and LV dilatation, and ameliorated LV systolic dysfunction as compared with that observed in the SD group. By contrast, LV dilatation and systolic dysfunction on LCD-A were more prominent than those on SD, and we noted further differences in LV dimensions and systolic function relative to those on LCD-P. Additionally, heart weight (HW) and the HW:tibial length (TL; HW/TL) ratio were lower in mice on LCD-P than in those on SD or LCD-A, which agreed with the result of LV mass (LVM) calculated using echocardiographic data (Fig. 1d–f). The expression levels of *Nppb,* a well-known marker gene for hypertrophy and heart failure, were also decreased in the heart of LCD-P-fed mice more than that of SD- or LCD-A-fed mice (Fig. 1g).

**Figure 2:**
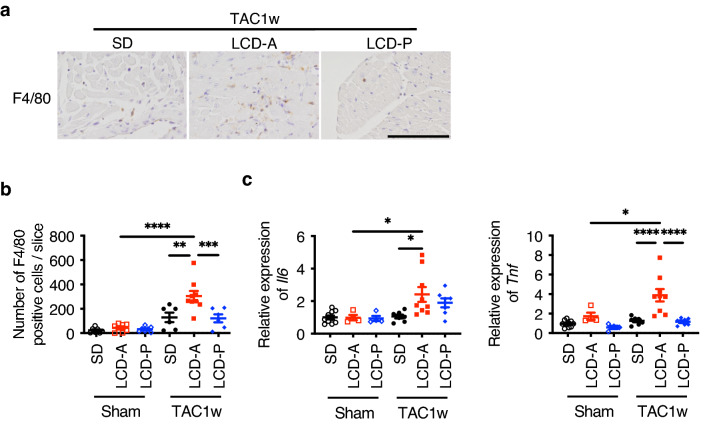
Effects of LCDs on cardiac inflammation induced by TAC surgery. **(a)** Representative immunofluorescent staining of heart sections with anti-F4/80 antibody at 1-week post-TAC surgery. Scale bar, 100 μm. (**b)** The number of F4/80-positive cells in heart sections at 1-week post-TAC or post-sham surgery (*n* = 6–9). (**c)** mRNA levels of *Il6* and *Tnf* in the heart at 1-week post-TAC or post-sham surgery (*n* = 4–10). Data represent the mean ± SEM. Statistical significance was analyzed by two-way ANOVA, followed by Holm–Sidak’s post-hoc test. *P < 0.05, **P < 0.01, ***P < 0.001, ****P < 0.0001.

Histochemical analyses revealed that LCD-A increased the number of inflammatory cells, such as F4/80-positive macrophages, in hypertrophied hearts at 1-week post-TAC, which was not observed in the SD or LCD-P groups (Fig. 2a,b). Moreover, LCD-A strongly upregulated the expression of inflammatory cytokines, such as *Il6* and *Tnf*, which was not observed in the SD or LCD-P groups (Fig. 2c). These results indicated that LCD-P and LCD-A exerted distinct effects on inflammation and HF progression.

### LCD-P increases the expression levels of PPARα target genes in hearts

**Figure 3:**
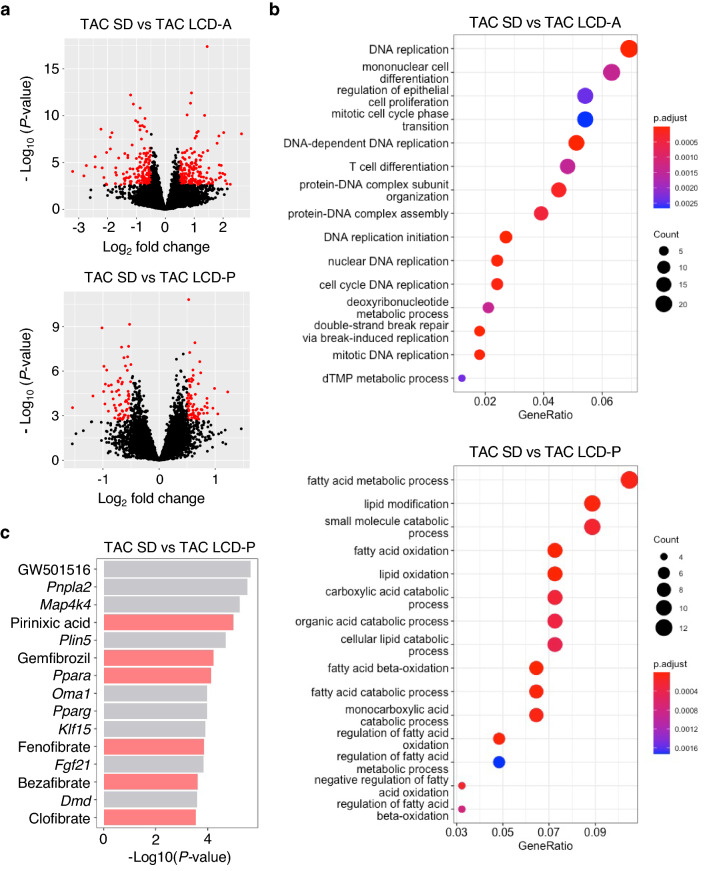
RNA-seq results in the hearts of mice fed different LCDs. **(a)** Volcano plot showing differentially expressed genes (DEGs) in the hearts of mice fed SD, LCD-A, or LCD-P diets at 1-week post-TAC surgery. (**b)** Gene Ontology analysis of DEGs between SD-fed mice and LCD-A- or LCD-P-fed mice after TAC surgery. (**c)** Top 15 upstream regulators of DEGs between SD-fed mice and LCD-P-fed mice after TAC surgery that were identified by Ingenuity Pathway Analysis. The red bar shows PPARα-related regulators.

**Figure 4:**
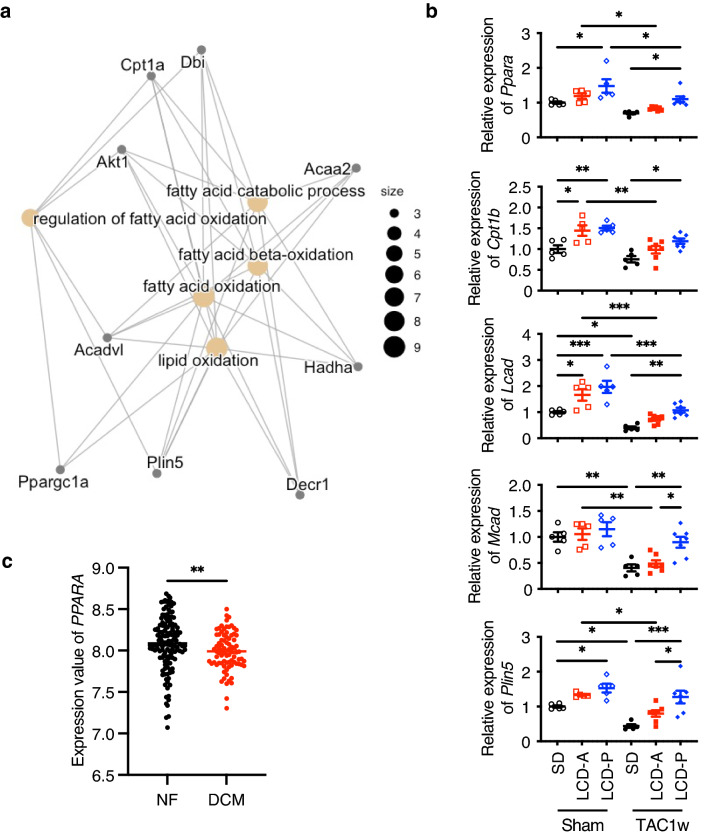
PPARα expression in failing hearts in mice and humans. **(a)** Significantly interacting genes among DEGs found in LCD-P-fed mice after TAC surgery. (**b)** mRNA levels of PPARα target genes in the heart at 1-week post-TAC or post-sham surgery (*n* = 5–7). (**c)** *PPARA*-expression levels in 136 non-failing (NF) hearts and 82 failing hearts with dilated cardiomyopathy (DCM), as determined by analyzing human Gene Expression Omnibus dataset GSE57338. Data represent the mean ± SEM. Statistical significance was analyzed by two-way ANOVA, followed by Holm–Sidak’s post-hoc test among multiple group comparisons. The Mann–Whitney *U* test was used for two-group comparisons. *P < 0.05, **P < 0.01, ***P < 0.001.

To elucidate the mechanisms by which LCD-P exerts its beneficial effects on pressure-overloaded hearts, we examined gene expression by RNA-sequencing (RNA-seq) analysis, which detected 14,683 genes. Using a false discovery rate of < 0.05 and an absolute $\sqrt{2}$ -fold change expression as cut-offs, we identified 363 differentially expressed genes (DEGs) in TAC mouse hearts in the LCD-A group from those in the SD, and that LCD-P altered the expression of 139 genes relative to the SD group (Fig. 3a and Supplementary Tables 1 and 2). We obtained the top 15 Gene Ontology (GO) terms from the enrichment analysis of these DEGs (Fig. 3b and Supplementary Tables 3 and 4). LCD-A induced the expression of cell cycle-related genes, whereas FAO-related genes were upregulated in LCD-P hearts. We then used Ingenuity Pathway Analysis (IPA) to elucidate the upstream regulators of the DEGs. IPA identified *Ppara*, a regulator of FAO, as the most highly activated transcription factor among the DEGs in LCD-P hearts (Fig. 3c and Supplementary Table 5). Furthermore, five PPARα agonists, including pirinixic acid, gemfibrozil, and fenofibrate, were detected among the top 15 potential upstream regulators. Additionally, network analysis of the DEGs indicated that PPARα target genes play critical roles in the hearts of mice on LCD-P (Fig. 4a). We subsequently confirmed upregulated expression of PPARα target genes (such as *Cpt1b, Lcad, Mcad*, and *Plin5*) using quantitative reverse transcription polymerase chain reaction (RT-qPCR) (Fig. 4b). Interestingly, even after TAC, the expression levels of some PPARα-related genes were clearly upregulated in LCD-P hearts, which was not observed in LCD-A hearts. Moreover, the Gene Expression Omnibus (GEO) dataset GSE57338 revealed that *PPARA* expression is downregulated in human hearts with HF, especially related to dilated cardiomyopathy (Fig. 4c). These results suggested that LCD-P enhances FAO through PPARα activation, leading to increased energy efficiency in the failing heart.

### Cardiac function is preserved by PPARα agonist and deteriorated by the loss of PPARα under stress

**Figure 5:**
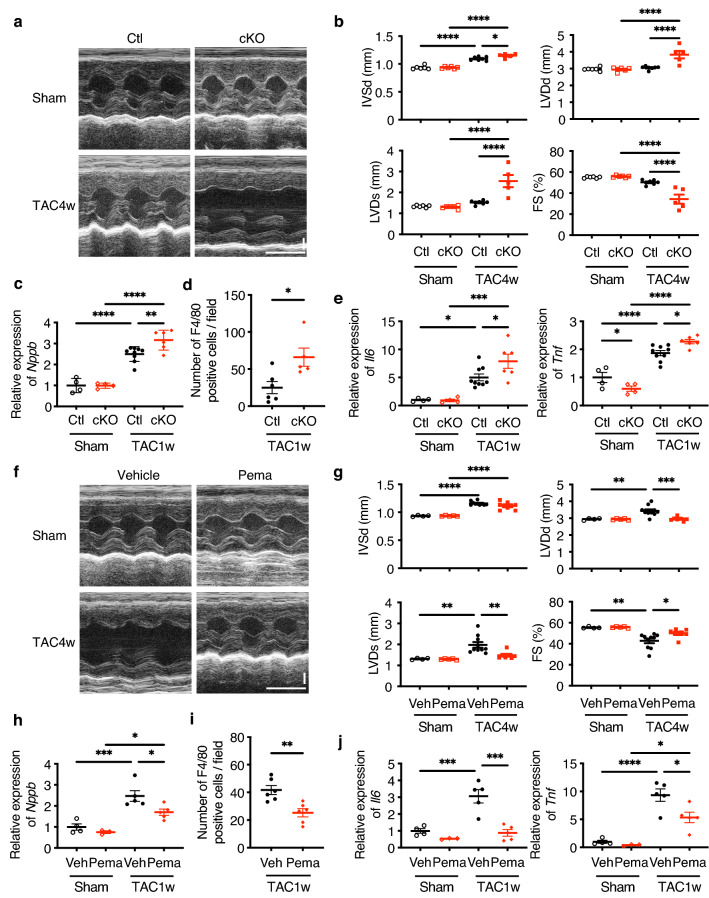
Effects of PPARα agonist and *Ppara* deletion on cardiac function. **(a)** Representative motion-mode (M-mode) echocardiography images of *Ppara*-conditional-knockout (cKO) mice at 4-weeks post-TAC or post-sham surgery. Vertical scale bar, 1 mm. Transverse scale bar, 100 ms. (**b)** Cardiac function of cKO mice, as assessed by M-mode echocardiography at 4-weeks post-TAC or post-sham surgery (*n* = 5–7). (**c)** mRNA level of *Nppb* in the hearts of cKO mice at 1-week post-TAC or post-sham surgery (*n* = 4–9). (**d)** The number of F4/80-positive cells per field of view in the hearts of cKO mice at 1-week post-TAC surgery (*n* = 5–6). (**e)** mRNA levels of *Il6* and *Tnf* in the hearts of cKO mice at 1-week post-TAC or post-sham surgery (*n* = 4–9). (**f)** Representative M-mode echocardiography images of mice in the pemafibrate group at 4-weeks post-TAC or post-sham surgery. Vertical scale bar, 1 mm. Transverse scale bar, 100 ms. (**g)** Cardiac function of mice in the pemafibrate group, as assessed by M-mode echocardiography at 4-weeks post- TAC or post-sham surgery (*n* = 4–10). (**h)** mRNA level of *Nppb* in the hearts of mice in the pemafibrate group at 1-week post-TAC or post-sham surgery (*n* = 3–5). (**i)** The number of F4/80-positive cells per field of view in the hearts of mice in the pemafibrate group at 1-week post-TAC surgery (*n* = 6). (**j)** mRNA levels of *Il6* and *Tnf* in the hearts of mice in the pemafibrate group at 1-week post-TAC or post-sham surgery (*n* = 3–5). Data represent the mean ± SEM. Statistical significance was analyzed by two-way ANOVA, followed by Holm–Sidak’s post-hoc test among multiple group comparisons. The two-tailed unpaired Student’s* t* test was used for two-group comparisons. *P < 0.05, **P < 0.01, ***P < 0.001, ****P < 0.0001.

Since we suspected that PPARα plays a central role in LCD-P-mediated cardioprotection against pressure overload, we investigated the effects of PPARα on HF. First, we examined the role of PPARα in HF development using cardiomyocyte-specific *Ppara*-conditional knockout (cKO) mice. Although there was no change in LV size or function in control and cKO mice without pressure overload, LV dimension was larger and LV systolic function was lower in cKO mice after TAC as compared with control mice (Fig. 5a,b). These physiological changes were accompanied with higher *Nppb* levels in cKO heart than control after TAC, indicating heart failure status in cKO (Fig. 5c). Additionally, we found a higher number of inflammatory cells and elevated gene-expression levels of inflammatory cytokines in cKO mice relative to those in control mice (Fig. 5d,e). We then activated PPARα using pemafibrate, a selective PPARα modulator, and found that pemafibrate ameliorated the TAC-induced LV dilatation and systolic dysfunction (Fig. 5f,g) and reduced *Nppb* gene expression (Fig. 5h). Furthermore, pemafibrate significantly decreased the number of infiltrating inflammatory cells and reduced the gene-expression levels of inflammatory cytokines in hypertrophied hearts (Fig. 5i,j).

### Loss of PPARα abolishes the cardioprotective effects of LCD-P

**Figure 6:**
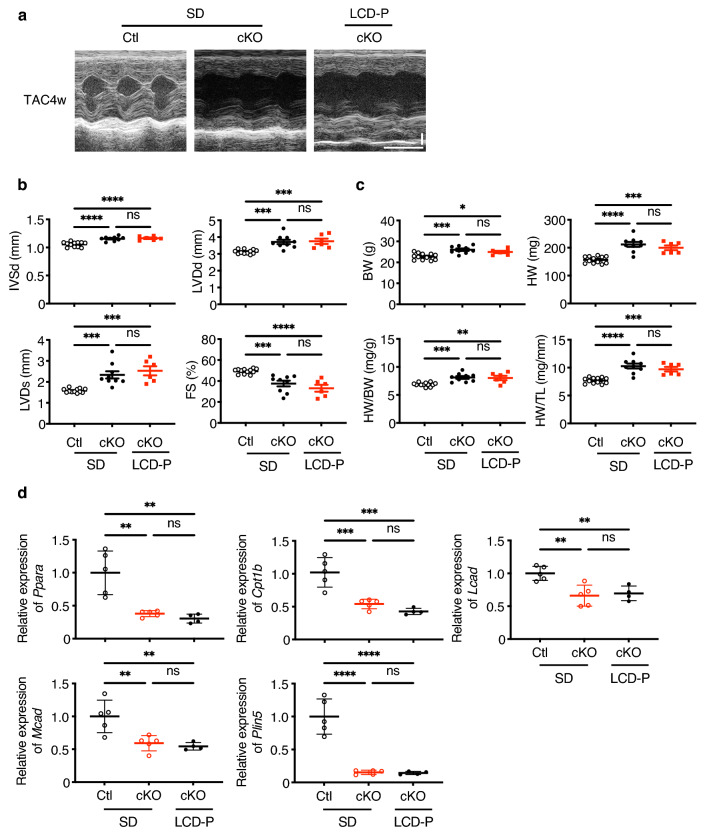
Effects of LCD-P under *Ppara* deletion on cardiac hypertrophy. **(a)** Representative motion-mode (M-mode) echocardiography images of *Ppara*-conditional-knockout (cKO) mice at 4-weeks post-TAC after 4-weeks of LCD-P feeding. Vertical scale bar, 1 mm. Transverse scale bar, 100 ms. (**b)** Cardiac function of cKO mice after 4-weeks of LCD-P feeding, as assessed by M-mode echocardiography at 4-weeks post-TAC surgery (*n* = 6–12). (**c)** Body weight (BW), heart weight (HW), HW:BW ratio, and HW:tibial length (TL) ratio at 4-weeks post-TAC surgery (*n* = 6–12). (**d)** mRNA levels of *Ppara* and its target genes in the hearts of cKO mice after 1-week of LCD-P feeding (*n* = 4–6). Data represent the mean ± SEM. Statistical significance was analyzed by two-way ANOVA, followed by Holm–Sidak’s post-hoc test. *P < 0.05, **P < 0.01, ***P < 0.001, ****P < 0.0001.

We then investigated whether LCD-P exerts beneficial effects on the heart via PPARα activation by using PPARα-cKO mice. In SD-fed cKO mice, TAC-induced cardiac hypertrophy and heart failure were observed as indicated by the increase in diastolic interventricular-septum thickness (IVSd), LV end-diastolic dimension (LVDd) and HW:body weight (BW; HW/BW) ratio and the decrease in LV fractional shortening (FS). In LCD-P-fed cKO mice, there were no significant differences in IVSd, LVDd, HW/BW, or FS compared to that of SD-fed cKO mice (Fig. 6a–c). Likewise, the expression levels of PPARα and its target gene expressions, which were upregulated by LCD-P in the wild mice (Fig. 4b), were unchanged by LCD-P in cKO mice (Fig. 6d), suggesting that the loss of cardioprotective effects of LCD-P in cKO heart were caused by the PPARα inactivity. Taken together, LCD-P exerts beneficial effects on the heart via PPARα activation.

### Stearic acid increases the expression levels of PPARα target genes

**Figure 7:**
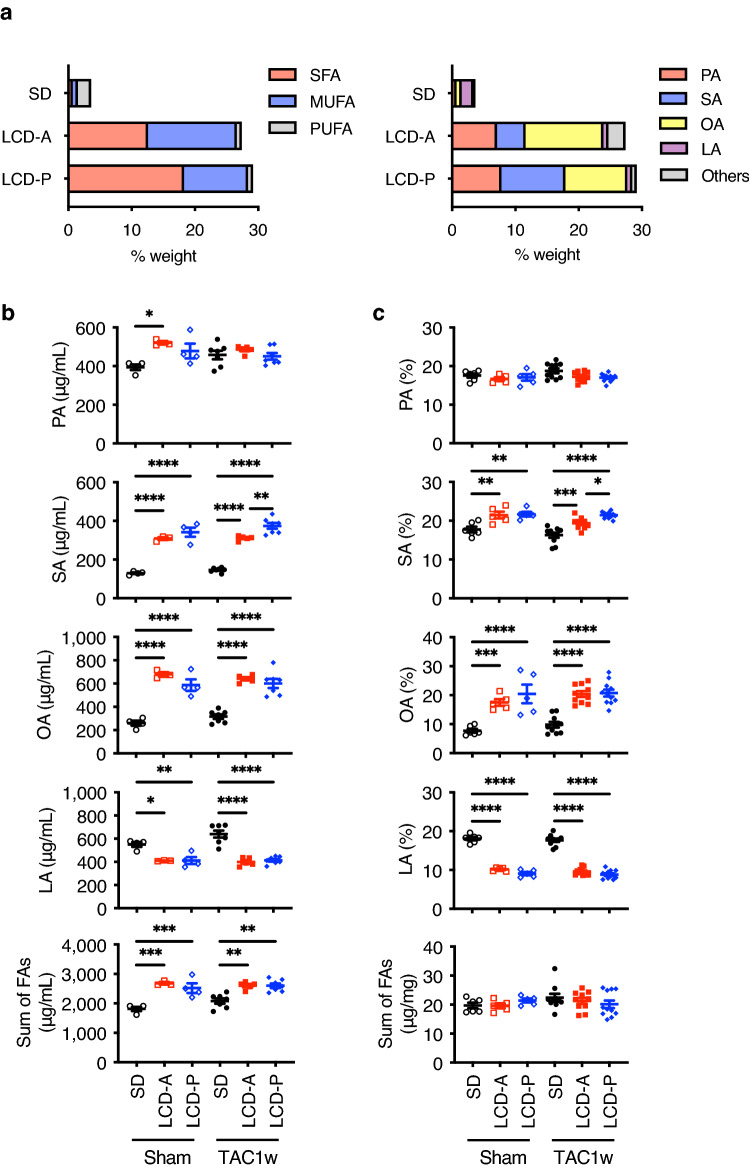
Selected FA compositions in the sera and hearts of mice. **(a)** FA profiles of total lipids among the three diet groups. LA, linoleic acid; MUFA, monounsaturated fatty acid; OA, oleic acid; PA, palmitic acid; PUFA, polyunsaturated fatty acid; SA, stearic acid; SFA, saturated fatty acid. (**b)** Selected FA compositions in the sera at 1-week post-TAC or post-sham surgery (*n* = 3–7). (**c)** Selected FA compositions in the hearts at 1-week post-TAC or post-sham surgery (*n* = 5–11). Data represent the mean ± SEM. Statistical significance was analyzed by two-way ANOVA, followed by Holm–Sidak’s post-hoc test. *P < 0.05, **P < 0.01, ***P < 0.001, ****P < 0.0001.

To elucidate the mechanism by which LCD-P activates PPARα, we first examined the fatty acid (FA) compositions of LCD-P and LCD-A. LCD-P was rich in saturated fatty acids (SFAs) and had much higher levels of stearic acid (SA) than LCD-A (Fig. 7a). By contrast, LCD-A had a much higher content of monounsaturated fatty acids (MUFAs) and oleic acid (OA) than LCD-P. Additionally, we examined the changes in FA profiles in the sera and hearts of mice at 1-week post-surgery, when there was no difference in cardiac function among the three groups. Each LCD resulted in a significant elevation of both serum SA and OA levels and reduced serum linoleic acid (LA) levels (Fig. 7b). Moreover, we observed a significant increase only in serum SA levels in LCD-P-fed mice as compared with those in LCD-A-fed mice, with similar patterns observed in SA, OA, and LA levels in the hearts of the respective mice (Fig. 7c). Although serum palmitic acid (PA) levels were higher in LCD-fed mice than in SD-fed mice, we found no significant difference between the LCD-A and LCD-P groups, and cardiac PA concentrations were similar among all three groups either with or without TAC. These findings suggested that LCD-P rich in SA might exert beneficial effects on hypertrophied hearts.

**Figure 8:**
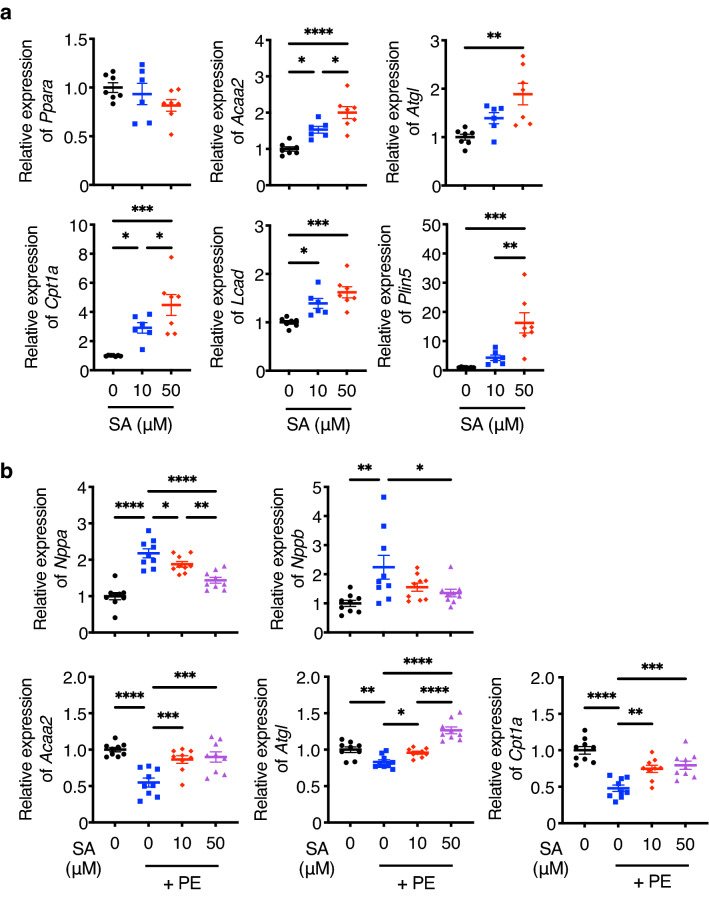
Expression levels of PPARα target genes in SA-stimulated cardiomyocytes. **(a)** mRNA levels of *Ppara* and its target genes in neonatal rat cardiomyocytes (NRCMs) after stimulation with SA for 6 h (*n* = 6–7). The SA concentrations tested were 10 μM and 50 μM. (**b)** mRNA levels of hypertrophic markers and PPARα target genes under SA stimulation in NRCMs treated with 100 μM phenylephrine (PE) for 24 h (*n* = 9). The SA concentrations tested were 10 μM and 50 μM. Data represent the mean ± SEM. Statistical significance was analyzed by one-way ANOVA, followed by the Holm–Sidak’s post-hoc test. *P < 0.05, **P < 0.01, ***P < 0.001, ****P < 0.0001.

Long-chain FAs, such as OA, PA, and SA, are physiological ligands of PPARα^19^. Thus, we added SA to the culture medium of neonatal rat cardiomyocytes (NRCMs). We identified SA-mediated elevations in the expression levels of PPARα target genes, such as *Acaa2, Atgl, Cpt1a, Lcad*, and *Plin5*, in a dose-dependent manner (Fig. 8a). Additionally, administration of phenylephrine (PE) reduced the expression levels of PPARα target genes, whereas this response was countered by SA stimulation (Fig. 8b). Consequently, SA reduced the expression levels of hypertrophy and heart failure marker genes *Nppa* and *Nppb* that were increased by PE. These results suggested that SA, which is abundant in the hearts of LCD-P-fed mice, plays an important role in cardioprotection by activating PPARα under the hypertensive state.

## Discussion

The results of recent cohort studies and meta-analyses suggest the importance of replacing carbohydrates in LCDs with fat and protein sources^12–14^. In the present study, we found that LCD-P ameliorated HF progression while LCD-A aggravated cardiac dysfunction and that the distinct effects of the LCDs on HF may depend on PPARα activation.

PPARα is a transcription factor abundantly expressed in the heart and regulates various physiological processes^16,20^. Its expression is downregulated in failing human and rodent hearts^21–23^, whereas mechanical unloading in failing human hearts increases the expression levels of PPARα and its target genes^24^. Global *Ppara*-KO mice demonstrate exacerbated cardiac dysfunction in response to pressure overload^25,26^, suggesting that PPARα is involved in HF development. Because PPARα is highly expressed in many other organs, including the liver, kidney, and skeletal muscle^15,27^, it is difficult to exclude any indirect effects of non-cardiac PPARα signaling on the heart. Furthermore, the effects of PPARα agonists on cardiac remodeling are controversial^22,28–30^. Therefore, to elucidate the role of cardiac PPARα, we generated cardiomyocyte-specific *Ppara*-cKO mice. Although there was no change in LV size and function in either control or cKO mice without pressure overload, cKO mice demonstrated more severe LV dysfunction and inflammation than control mice following TAC (Fig. 5b–e). Additionally, PPARα activation by pemafibrate, a selective PPARα modulator, ameliorated LV dysfunction and TAC-induced inflammation (Fig. 5g–j). The activation ability and selectivity of fibrates previously used as PPARα agonists might be low, and they could activate other PPAR isotypes, such as PPARγ and PPARδ, to some extent. By contrast, pemafibrate is a more potent and selective PPARα agonist than other fibrates^31–33^. The results of the loss- and gain-of-function experiments in the present study clearly indicated that PPARα activation was beneficial in preventing HF development and that the cardioprotective effects of LCD-P were PPARα dependent (Fig. 6b–d).

Cardiac hypertrophy accelerates the metabolic-flux shift from FAO to glucose oxidation, which may lead to energy insufficiency^34^. Several studies have shown improvement in cardiac hypertrophy and function by enhancing FAO^35–37^. Since PPARα reportedly activates FAO, LCD-P might exert beneficial effects on cardiac function in the presence of pressure overload by activating FAO via PPARα activation in order to meet the high cardiac energy demand^16^. In the present study using RNAseq, among 139 differentially expressed genes between SD and LCD-P, we found FAO-related genes being upregulated in LCD-P. Further analysis for upstream regulators of FAO revealed *Ppara* as the most highly activated transcription factor. Since the depletion of *Ppara,* specifically in cardiomyocytes, abolished the beneficial effects of LCD-P and the upregulation of FAO-related genes, we suggest that a possible mechanism by which LCD-P protects the heart is the enhancement of FAO through PPARα activation, leading to increased energy efficiency in the failing heart.

Inflammation reportedly causes cardiac dysfunction, and PPARα decreases the expression of proinflammatory genes such as *Il6* and *Tnf*^15,38^. The present study exhibiting the exacerbation of cardiac inflammation by LCD-A but not by LCD-P suggested that LCD-P may have the potential to prevent the development of cardiac dysfunction by PPARα-mediated suppression of inflammation. Indeed, the PPARα cKO heart demonstrated a higher number of F4/80 positive inflammatory cells in the TAC heart (Fig. 5d) and, conversely, pemafibrate, a selective PPARα activator, successfully reduced the inflammation (Fig. 5i). These results suggest that LCD-P ameliorated HF through PPARα-mediated anti-inflammatory effects.

X-ray crystallography recently revealed SA and PA as physiological PPARα ligands^39^. In the present study, the results showed that SA dose-dependently upregulated the expression of PPARα target genes in cultured cardiomyocytes in vitro and restored the expression of genes downregulated by PE, a cardiomyocyte hypertrophy-inducing factor^40^ (Fig. 8a,b). These results suggest that abundant SA in LCD-P enhances PPARα activity in hypertrophied hearts. Additionally, SA reportedly improves mitochondrial function by increasing mitochondrial fusion in *Drosophila* and humans^41,42^ and exerts an anti-inflammatory response in cholestatic liver injury by reducing leukocyte accumulation and NF-κB activity in rats^43^. Moreover, a previous study reported the neuroprotective effects of SA against oxidative stress in the rat brain^44^. These results collectively suggest that SA protects various organs by activating multiple pathways, including the PPARα signaling pathway as shown in the heart.

This study has limitations. First, we used only one type of fat source for LCD-A and LCD-P, respectively; therefore, further studies are required to confirm whether the results of this study are dependent on animal- or plant-derived fat. Second, we did not provide precise mechanisms related to worsening HF by LCD-A. Exacerbation of cardiac inflammation and the associated elevation of inflammatory genes were the hallmarks of LCD-A-fed heart tissue, but no possible upstream regulators of these changes were discovered even with RNA-seq analysis. Well-used high-fat diets often consist of animal fats such as lard, which are similar to LCD-A and are associated with poorer cardiac outcomes during stress or aging^45–47^. Third, we could not measure the FAO or glucose oxidation rate, which remains to be clarified in future research. Finally, there are controversies in the relationship between SFA and CVD^48^. Given that individual SFAs have different biological effects and their long-term influence on health remains unclear, the duration, timing, and amount of LCDs containing plant-derived fat should be carefully optimized before clinical application.

In conclusion, these findings suggest that substituting reduced carbohydrates with plant-derived fat is beneficial to preventing HF development and that the LCD-P-SA-PPARα pathway may be a potential therapeutic target for treating HF.

## Methods

### Mice

All animal experiments were approved by the Ethics Committee for Animal Experiments of the University of Tokyo (Tokyo, Japan) and adhered strictly to animal experimental guidelines and the ARRIVE guidelines. The procedures strictly adhered to the guidelines for animal experiments of the University of Tokyo and the National Institutes of Health guidelines for the Care and Use of Laboratory Animals. All mice were housed under a controlled temperature with a 12-h light/dark cycle and provided food and water ad libitum.

For the LCD experiments, 9- to 10-week-old male C57BL/6J mice were purchased from CLEA Japan (Tokyo, Japan). Littermate mice of similar ages and weights were randomized to experimental and control groups. The mice were anesthetized with 2% isoflurane and subjected to TAC (26-gauge needle) or sham surgery, as previously described^49^. After the surgery, cages of the mice were randomized to the feeding protocols by another researcher who was blinded to the experiments and those analyses. They were fed either a standard diet (CE-2; 59% carbohydrate and 12% soybean-based fat of total energy, 344 kcal/100 g; CLEA Japan), an LCD-A diet (12% carbohydrate and 59% beef tallow-based fat of total energy, 485 kcal/100 g; Oriental Yeast, Tokyo, Japan), or an LCD-P diet (12% carbohydrate and 59% cocoa butter-based fat of total energy, 485 kcal/100 g; Oriental Yeast). The mice were fed the indicated diets ad libitum for 4 weeks starting from the day of surgery.

Pemafibrate was kindly provided by Kowa (Aichi, Japan). Specifically, 9- to 10-week-old male C57BL/6J mice received pemafibrate (1.0 mg/kg body weight) orally daily from 1 week before TAC (26-gauge needle) or sham surgery until the end of the study. Methylcellulose (0.5%) was used as the vehicle.

*Ppara*-cKO mice were generated using the Cre/loxP system. *Ppara*^*flox/flox*^ mice were provided by Dr. Frank J. Gonzalez (National Cancer Institute, Bethesda, MD, USA)^50^. *αMHC-Cre* mice (Tg [Myh6-cre] 1Jmk/J, #009074) were purchased from the Jackson Laboratory (Bar Harbor, ME, USA). Specifically, 9- to 10-week-old male *αMHC-Cre*^+*/−*^; *Ppara*^*flox/flox*^ mice underwent TAC (25-gauge needle) or sham surgery, with littermate control mice (*Ppara*^*flox/flox*^) operated in the same way. As *αMHC-Cre*^+*/−*^; *Ppara*^*flox/flox*^ mice were more vulnerable to TAC-induced heart failure and died soon after the surgery, we used a larger needle (25-gauge), not a standard one (26-gauge), leaving milder aortic constriction.

The mice were analyzed at 1- and 4-weeks post-surgery. Transthoracic echocardiography was performed on conscious mice using a Vevo2100 ultrasound system (FUJIFILM VisualSonics, Toronto, ON, Canada). Diastolic interventricular-septum thickness (IVSd), diastolic posterior-wall thickness (PWd), LV end-diastolic dimension (LVDd), and LV end-systolic dimension (LVDs) measurements were taken using motion-mode echocardiography. LV fractional shortening (FS) was calculated as follows: FS = (LVDd − LVDs)/LVDd × 100. LVM was calculated as follows: 1.05 × {(IVSd + LVDd + PWd)^3^ − (LVDd)^3^}. Blood samples were collected by intracardiac puncture under 2% isoflurane anesthesia. Deeply anesthetized mice were euthanized by cervical dislocation. Serum samples were isolated by centrifugation at 3000 rpm for 15 min.

### Immunohistochemical analysis

Mouse hearts were fixed with 20% formalin (Sakura Finetek Japan, Tokyo, Japan), embedded in paraffin, and sectioned at a 4-μm thickness. The sections were stained with anti-F4/80 antibodies (MCA497GA; Bio-Rad, Hercules, CA, USA) to evaluate cell infiltration. The number of infiltrating cells was counted in each heart section for the LCD experiments and in each visual field (200×) for the pemafibrate and *Ppara*-cKO mouse experiments. Visual fields were selected randomly under a BZ-X800 microscope (Keyence, Osaka, Japan), and the average number of infiltrating cells in five fields was calculated for each sample.

### RT-qPCR analysis

Total RNA was extracted from cells and tissues using an RNeasy Mini kit (Qiagen, Hilden, Germany) or a tissue total RNA Mini kit (Favorgen, Ping-Tung, Taiwan), respectively, according to manufacturer instructions. cDNA was generated using ReverTra Ace qPCR RT master mix (Toyobo, Osaka, Japan), and qPCR was performed using Thunderbird Next SYBR qPCR mix (Toyobo) with primers specific for each gene of interest (Supplementary Table 6). The resulting data were analyzed using a QuantStudio 5 instrument (Thermo Fisher Scientific, Waltham, MA, USA). Relative gene expression levels were determined using the relative standard curve method and normalized against the expression of 18S ribosomal RNA.

### Bulk RNA-seq analysis

Total RNA was extracted from mouse heart tissues at 1-week post-TAC or post-sham surgery (*n* = 3 mice/group) and used to generate RNA-seq libraries with a TruSeq stranded mRNA library prep kit (Illumina, San Diego, CA, USA). The Illumina HiSeq 2500 platform was used for sequencing. Raw reads were checked for quality using the FastQC program (version 0.11.15) and trimmed using Trimmomatic (version 0.36), where low-quality bases with Phred quality scores of < 33 were discarded. Adaptor sequences were removed using Cutadapt (version 1.14). STAR aligner (version 2.5.2b) was used to align clean reads to the mouse reference genome (mm9). RSubread-2.0.1-FeatureCounts software was employed for quantification, and DESeq2 software (version 1.28.0) was used for differential analysis using R software (version 3.5.1).

### GEO dataset analysis

We selected the GSE57338 dataset to analyze differences in gene expression associated with human HF. *PPARA* expression levels were compared in 136 non-failing hearts and 82 failing hearts with dilated cardiomyopathy. The gene expression levels were determined using the GEO2R web tool.

### FA analysis

Dietary FAs were measured by Japan Food Research Laboratories (Tokyo, Japan) using gas chromatography-mass spectrometry (GC–MS), as previously described^51^.

The FA compositions in serum and heart samples were measured by a central laboratory (BML, Tokyo, Japan). Lipids from the hearts were extracted as previously described by Folch et al.^52^ After spiking the samples with tricosanoic acid as an internal control, serum and heart lipids were methylated using boron trifluoride and methanol. The methylated FAs were then measured by GC–MS analysis (QC-2010; SHIMADZU, Kyoto, Japan)^53^.

### NRCM culture

NRCMs were prepared as previously described^54^. NRCMs were obtained from 0- to 2-day-old Wistar rats (Takasugi Experimental Animal Supply, Saitama, Japan), dispersed by collagenase digestion, and subjected to Percoll gradient centrifugation. Isolated cardiomyocytes were cultured for 48 h in Dulbecco’s modified Eagle medium (Nacalai Tesque, Kyoto, Japan) supplemented with 10% fetal calf serum. After 24 h of serum starvation, NRCMs were stimulated with SA and PE.

### SA stimulation

SA (S4751; Sigma-Aldrich, St. Louis, MO, USA) was conjugated to FA-free bovine serum albumin (BSA; A6003; 20% concentration; Sigma-Aldrich) at a molecular ratio of 2.2:1. Working stocks of SA (10 μM or 50 μM; used for stimulation) were prepared from a 5 mM stock solution. NRCMs were treated with SA for 6 h in the absence of PE. Cardiomyocyte hypertrophy was induced by treatment with 100 μM PE (P6126; Sigma-Aldrich) for 24 h. In the presence of PE, NRCMs were pre-stimulated with SA 2 h before PE treatment. Control cells were cultured in the presence of 0.03% BSA alone.

### Statistical analysis

Data are shown as mean ± standard error of the mean (SEM). Statistical analyses were performed using GraphPad Prism software (version 9.2.0; GraphPad Software, San Diego, CA, USA). A two-tailed unpaired Student’s *t*-test or a Mann–Whitney *U* test was performed to compare the two groups. One-way or two-way ANOVA followed by Holm-Sidak’s post-hoc analysis was conducted to compare multiple groups. The threshold for statistical significance was set at *P* < 0.05.

## Supplementary Information

Supplementary Information.

## Data Availability

The data underlying this article will be shared on reasonable request to the corresponding author.
