# Supplementary material for: Low-carbohydrate diets containing plant-derived fat but not animal-derived fat ameliorate heart failure
Source: Sci Rep. 2023 Mar 9;13:3987. doi: 10.1038/s41598-023-30821-7 (PMC9998649; doi:10.1038/s41598-023-30821-7)
Supplement: Supplementary file 1 — Supplementary Information. [file 41598_2023_30821_MOESM1_ESM.pdf]

Low-carbohydrate diets containing plant-derived fat but not animal-derived fat ameliorate heart failure

Satoshi Bujo[1,2], Haruhiro Toko[1*], Kaoru Ito[3], Satoshi Koyama[3], Masato Ishizuka[1], Masahiko Umei[1], Haruka Yanagisawa-Murakami[1], Jiaxi Guo[1], Bowen Zhai[1], Chunxia Zhao[1], Risa Kishikawa[1], Norifumi Takeda[1], Kensuke Tsushima[1], Yuichi Ikeda[1,2], Eiki Takimoto[1], Hiroyuki Morita[1], Mutsuo Harada[1,4], Issei Komuro[1*]

[1] Department of Cardiovascular Medicine, Graduate School of Medicine, The University of Tokyo; Bunkyo-ku, Tokyo 113-8655, Japan

[2] Department of Advanced Translational Research and Medicine in Management of Pulmonary Hypertension, Graduate School of Medicine, The University of Tokyo; Bunkyo-ku, Tokyo 113-8655, Japan

[3] Laboratory for Cardiovascular Genomics and Informatics, RIKEN Center for Integrative Medical Sciences; Tsurumi-ku, Yokohama, Kanagawa 230-0045, Japan

[4] Department of Advanced Clinical Science and Therapeutics, Graduate School of Medicine, The University of Tokyo; Bunkyo-ku, Tokyo 113-8655, Japan

Supplementary information

Supplementary Table 1. DEGs of TAC mice hearts between SD and LCD-A: Page 2-10

Supplementary Table 2. DEGs of TAC mice hearts between SD and LCD-P: Page 11-14

Supplementary Table 3. GO analysis data of TAC mice hearts between SD and LCD-A: Page 15-18

Supplementary Table 4. GO analysis data of TAC mice hearts between SD and LCD-P: Page 19-20

Supplementary Table 5. IPA data of TAC mice hearts between SD and LCD-P: Page 21-31

Supplementary Table 6. Sequences of the primers used for RT-qPCR: Page 32

Supplementary Figure 1. Food intake and serum triglyceride levels among diet groups: Page 33

Supplementary Table 1. DEGs of TAC mice hearts between SD and LCD-A

gene	log2FoldChange	pvalue	adjusted pvalue
Mcm2	1.44806404	4.18E-18	5.90E-14
Tuba1b	0.90462694	3.74E-13	2.64E-09
Tecrl	-1.2040106	6.21E-13	2.92E-09
Actb	0.87193973	4.63E-12	1.63E-08
Sult1a1	-1.107468	5.82E-12	1.64E-08
Art3	-0.8737175	1.54E-11	3.63E-08
Actg1	0.75281504	8.29E-11	1.64E-07
Mcm3	1.35915583	9.31E-11	1.64E-07
Thnsl2	-0.7055768	1.98E-10	3.11E-07
Fmo5	-1.0309093	3.38E-10	4.76E-07
Dpep1	-0.9548035	4.88E-10	6.26E-07
LOC102636514	-0.7979552	1.14E-09	1.34E-06
Ppip5k2	-0.8801611	1.23E-09	1.34E-06
Scara5	-0.727569	1.55E-09	1.56E-06
Lvrn	-2.2314611	2.76E-09	2.60E-06
Abat	-0.8782682	3.25E-09	2.87E-06
Agtpbp1	-0.6432437	4.72E-09	3.71E-06
Prn	1.14023838	4.73E-09	3.71E-06
Ccnd1	1.08810794	6.34E-09	4.44E-06
Apln	2.02051494	6.37E-09	4.44E-06
Sema5a	-1.8495011	6.61E-09	4.44E-06
Gdf15	2.63712957	8.66E-09	5.56E-06
Mcm5	1.85338271	1.48E-08	8.62E-06
Mcm6	1.07280825	1.53E-08	8.62E-06
Pbld1	-1.9065152	2.00E-08	1.08E-05
Spry4	1.02711008	2.48E-08	1.30E-05
Rgs2	-0.8294098	3.13E-08	1.58E-05
Aqp8	1.79236908	8.79E-08	4.28E-05
4732416N19Rik	-1.1504593	1.35E-07	6.34E-05
Cript	-0.5260802	2.93E-07	0.000130186
Mturn	-0.7261677	3.11E-07	0.000132944
Plxnd1	0.88323181	3.24E-07	0.000134389
Stab2	-1.9047342	3.56E-07	0.000143423
Rcc1	0.74563654	5.24E-07	0.000199894
Tcf19	1.46460705	5.38E-07	0.000199894
Pigp	-0.5338674	6.16E-07	0.000223003
Hn1l	1.00977722	7.47E-07	0.000261798
Dcakd	0.66671375	8.40E-07	0.000261798
Ptma	0.68036722	8.45E-07	0.000261798
Spock2	-2.0365447	8.48E-07	0.000261798

Nes	1.30497704	8.53E-07	0.000261798
Nme1	0.5897623	9.29E-07	0.000271746
Pygo1	-0.6482989	9.43E-07	0.000271746
Nek7	-0.6127778	9.91E-07	0.000279662
Unc5a	0.93421893	1.02E-06	0.000283517
Phyhd1	-0.7422416	1.05E-06	0.000285188
Mgst1	-0.7395942	1.08E-06	0.00028788
Hif3a	-1.4052109	1.10E-06	0.00028788
3110057O12Rik	-0.8717524	1.16E-06	0.000293675
Abca6	-0.6648563	1.17E-06	0.000293675
Cops2	-0.5137813	1.69E-06	0.000403312
Scgb1c1	-1.2994642	1.70E-06	0.000403312
Lig1	1.16945726	1.74E-06	0.000403312
Gatsl2	-0.6679612	1.91E-06	0.000428519
Plk3	1.24614184	2.19E-06	0.000474604
Slc12a3	-2.4303216	2.42E-06	0.000509645
C920006O11Rik	-0.9003587	2.56E-06	0.000531245
Acss1	-0.5649942	2.69E-06	0.000549505
Ppm1k	-0.5542591	2.87E-06	0.000575403
Mtbp	0.83117729	2.90E-06	0.000575403
Ssbp4	0.60290329	2.94E-06	0.000575403
Sbk3	-1.300312	3.05E-06	0.000588881
Slc5a1	-1.6170399	3.30E-06	0.000621231
Ifi205	-0.8150912	3.66E-06	0.000679898
Tmtc1	-1.0042868	3.72E-06	0.000681756
Rrm1	0.65780973	4.07E-06	0.000735717
Mfap1a	-0.6245224	4.41E-06	0.000787208
Isyna1	-0.6671632	4.57E-06	0.000802569
Tubb6	0.68281816	4.61E-06	0.000802569
Shb	1.06370584	5.08E-06	0.000874074
Plaur	1.21173199	5.41E-06	0.000920317
Nae1	-0.5394816	5.88E-06	0.000988371
Ogdhl	-0.6781764	6.19E-06	0.001027257
Aldh6a1	-0.5464697	6.57E-06	0.001072658
Spc24	1.05856875	7.19E-06	0.001153691
Actn4	0.51953895	7.70E-06	0.001220277
Slc25a27	-0.6642483	8.28E-06	0.001284811
Slbp	0.54086465	9.26E-06	0.00138522
Tmem71	-0.8721488	9.32E-06	0.00138522
Ypel3	-0.7060519	9.64E-06	0.001389705
Tspan9	0.91996618	9.95E-06	0.001403852
Chpf	0.52736624	1.02E-05	0.00142386
Lpl	-0.5730926	1.15E-05	0.001572384

4930452B06Rik	0.88801079	1.15E-05	0.001572384
Gadd45g	1.03580468	1.17E-05	0.001589279
Vwa1	0.93131947	1.24E-05	0.001666973
Tubb5	0.73054723	1.33E-05	0.001751341
Csf1	0.90478789	1.34E-05	0.001751341
Mxi1	-0.5747935	1.38E-05	0.001783684
Traf3	0.5777148	1.56E-05	0.001998432
Ift122	1.07571539	1.74E-05	0.002168767
Timp4	-0.7433385	1.84E-05	0.002260432
Uhrf1	1.86651651	1.84E-05	0.002260432
Dnmt1	0.73521836	1.86E-05	0.002261764
Irf2bpl	0.53494552	1.99E-05	0.002375105
Ncapd2	0.79456284	2.02E-05	0.002394861
Arc	1.935745	2.04E-05	0.002402249
Inmt	-2.7382163	2.11E-05	0.002458421
Myh9	0.85440691	2.53E-05	0.002831778
4831440E17Rik	-0.8800721	2.55E-05	0.002831778
Ier2	0.98699748	2.64E-05	0.002903438
Pidd1	1.21412882	2.76E-05	0.002979466
Dpyd	-0.6201478	2.77E-05	0.002979466
Mcam	0.9915238	2.82E-05	0.002995528
Acta2	1.1712618	2.90E-05	0.003058871
Slc22a12	-2.4320284	2.95E-05	0.003076703
Pole	0.83641715	3.07E-05	0.003099442
Mettl20	-0.8342695	3.26E-05	0.003221256
Wdr45	-0.615466	3.29E-05	0.003226341
Mesdc1	0.87803105	3.34E-05	0.003239094
Ralb	0.5444479	3.37E-05	0.003239094
Ddx39	0.53647368	3.37E-05	0.003239094
Galnt15	-0.9265624	3.51E-05	0.003323333
Slc37a1	-0.5564985	3.58E-05	0.003348801
Dpysl5	-1.7172438	3.67E-05	0.003411524
Arrdc5	-2.1892156	3.72E-05	0.003427505
Asf1b	1.50682315	3.93E-05	0.003508696
Tubb2a	0.65819907	4.08E-05	0.003594785
Jade2	-0.6881716	4.13E-05	0.003623817
Junb	1.06868323	4.17E-05	0.003628588
Urm1	0.51839768	4.33E-05	0.003745925
Chaf1a	1.02335874	4.38E-05	0.003771516
Ppp1r14a	0.92381414	4.51E-05	0.003859669
Ace2	-0.9810307	4.55E-05	0.003866483
Hist1h1c	-1.2225699	4.60E-05	0.003889303
Tmem179	-1.1238606	4.95E-05	0.004102433

Aebp1	0.93876064	5.39E-05	0.004324381
Rasip1	0.51589917	5.51E-05	0.004394727
Cux2	0.63844469	5.64E-05	0.004471743
Gm16793	0.67403437	5.97E-05	0.004679851
Gimap9	0.97722557	6.05E-05	0.004692873
Nt5c3	-0.721075	6.20E-05	0.004755648
Zfp445	-0.5721853	6.27E-05	0.004784834
Pola1	0.8235971	6.32E-05	0.004791932
Klhl11	-0.7088785	6.63E-05	0.005002884
Flnc	0.58829137	7.02E-05	0.005235161
Cul4b	-0.5640905	7.05E-05	0.005235161
Map7	-0.9444856	7.74E-05	0.005692781
Slc25a25	0.82431155	8.36E-05	0.006053403
Paqr6	-3.2128114	8.98E-05	0.006434979
Arl4a	-0.5533294	9.31E-05	0.006604019
Akap5	1.1246611	9.37E-05	0.006610211
Zfp943	-0.7875374	9.92E-05	0.006967544
Wdhd1	1.20665683	0.00010608	0.007302941
Bnip3	-1.3349926	0.000107648	0.007374965
Fmo2	-1.722481	0.000115866	0.007749873
Megf11	1.4430968	0.000119849	0.007978451
Adam19	0.84369404	0.000125611	0.008322788
Chaf1b	1.88479693	0.000132944	0.008662869
Zfp229	-0.6240503	0.000133199	0.008662869
Tfrc	0.96095075	0.00013845	0.008922146
Egr1	1.98476044	0.00014516	0.009145733
Atad2	1.22285535	0.000148841	0.009297963
Xdh	-0.785894	0.000148894	0.009297963
Arhgap5	-0.5741688	0.000149636	0.009303168
Tgfb1i1	0.72096754	0.000150694	0.009327812
Ppp1r3a	-0.5192999	0.000151545	0.009339545
Lgals1	0.60602609	0.000153994	0.009449195
Trp53	0.66197308	0.000156068	0.009535029
Thbs1	1.18957671	0.000159001	0.009672305
Abcb1a	-0.5944556	0.000160394	0.00970355
Peg3os	-1.0758899	0.00016362	0.009764008
D630039A03Rik	-1.4560486	0.000164517	0.009764008
Wdr90	0.82677576	0.000164659	0.009764008
Egfl7	0.5376315	0.00016848	0.00994877
1010001N08Rik	-0.5014924	0.000179043	0.010484767
Camk2n1	-0.6216115	0.000188688	0.010900367
Ccl7	2.0582553	0.000190001	0.010900367
Smg9	0.52372577	0.000193483	0.011010591

Amigo2	-0.6546678	0.00019518	0.011043994
Arhgap20	-0.5141036	0.000197168	0.011043994
Myom2	-0.9315473	0.0001972	0.011043994
Trim23	-0.5976004	0.000200162	0.01116554
Rnase1	-1.6944449	0.00021869	0.01205614
Zfp800	-0.5920626	0.000219972	0.012073334
Pola2	0.57599916	0.000220713	0.012073334
Cchcr1	0.78368707	0.000228358	0.012443326
Zfp950	-0.5264695	0.000236102	0.012717943
Peg3	-0.9100302	0.000242177	0.012849033
Tmem19	-0.5008675	0.000245327	0.012967419
D830032E09Rik	-0.7651368	0.000255584	0.013261225
Lmnb2	1.16379805	0.000265044	0.013580657
Ints2	0.9279636	0.000265371	0.013580657
Apoa4	-2.822359	0.000266362	0.013580657
Bmp6	-0.5501135	0.000267855	0.01359797
1810055G02Rik	0.81988486	0.000272088	0.013763376
Tbx5	-0.8064522	0.000281779	0.014052095
Dysf	0.66225194	0.000295274	0.014419386
Csrp1	0.50770259	0.000315221	0.015183316
C330022C24Rik	-1.2205631	0.000324482	0.015560374
Prdm1	1.23893785	0.000328697	0.015631089
Zfp52	-0.7957751	0.000329657	0.015631089
Pcp4l1	-0.7672667	0.00033616	0.015866962
Pnpla7	-1.0260845	0.000342344	0.016104983
Fos	1.91851992	0.000347663	0.016250944
Crocc	0.67785502	0.000355347	0.016551218
Syn2	-0.819735	0.00035827	0.01663244
Adamts17	1.32272732	0.000362074	0.016657597
Apoo	-0.7325726	0.00036455	0.016704213
Cfh	-0.5563415	0.000366198	0.016723623
Arhgef19	0.60826582	0.000367344	0.016723623
H2afz	0.68564187	0.000373922	0.016913965
Trnp1	-0.9645641	0.000384992	0.017165884
Bcl2l1	-1.0281043	0.000386654	0.017165884
Naa30	-0.5225144	0.00039678	0.017433065
Cdo1	-2.1292112	0.000398738	0.017433065
Dll4	0.59391687	0.0004033	0.017513157
Ankrd23	0.71731468	0.000407431	0.017584303
Dtx1	-1.3512803	0.000425797	0.018260771
Slc10a6	-1.2686982	0.000427367	0.018260771
Cdc6	1.86828321	0.000443164	0.01855897
Gins2	1.42197696	0.00045665	0.019010933

Cyp26b1	-1.3735793	0.000458954	0.019031269
Serinc3	-0.5568591	0.000463903	0.019082037
Cdc37l1	-0.5001742	0.000467514	0.019124693
Alg6	-0.5766714	0.000477194	0.019408175
Nudt1	0.56852221	0.000479113	0.019430242
Cfl1	0.67810969	0.000490562	0.01978088
Dus4l	-0.5815531	0.000494153	0.019812465
Mfsd7c	-0.6763717	0.000501615	0.019829937
Trim16	0.55637212	0.000506768	0.01987832
Fen1	0.7266875	0.000507064	0.01987832
Gm14420	-0.6856388	0.00050888	0.019894235
Ttyh2	0.94366978	0.000519044	0.020179801
Mtfp1	0.60715368	0.000521838	0.020232679
Cd46	-1.4283979	0.000531664	0.020501032
Smarcb1	0.56553242	0.000545675	0.02080098
Tk1	1.57047157	0.000546812	0.02080098
Rbl2	-0.6727233	0.000605726	0.022629914
Flna	0.63975776	0.000606314	0.022629914
Asb11	-0.6054732	0.000607719	0.022629914
Rnpc3	-0.7772285	0.000610404	0.022635531
Apc2	0.88702933	0.000619466	0.022852083
Zrsr1	-0.5293649	0.000622016	0.022852083
Rnaseh2c	0.63079139	0.000640239	0.023348054
Zfp654	-0.568067	0.000645897	0.023493678
Tonsl	1.04327306	0.000664201	0.023974092
Scml4	1.60498831	0.000681948	0.024489399
Polr3h	-0.5013734	0.000689572	0.02464985
Dok2	0.98841265	0.000694698	0.024695679
Cytl1	-0.7325043	0.000695201	0.024695679
Ptbp1	0.55057493	0.000696442	0.024695679
Tyms	1.74573341	0.000707128	0.024949261
Nadk2	-0.5210797	0.000711973	0.024995222
Wfikkn2	-0.6124106	0.000718254	0.025090877
Iqcb1	-0.617043	0.000729703	0.02521686
Mustn1	-0.6096318	0.00076499	0.02607803
Dctd	0.99154251	0.000778653	0.026330831
Unc13a	-0.8062432	0.000779869	0.026330831
Gimap6	0.5157182	0.000782713	0.026359857
Sall2	0.93275407	0.000784464	0.026359857
Spata13	0.58655034	0.000794599	0.02659651
Vwa3a	-0.5317111	0.000795276	0.02659651
Lgalsl	-0.5190205	0.00082054	0.02731198
Crtap	0.57668448	0.000824197	0.027369154

Fam117b	-0.6446934	0.00083227	0.027531492
Tsc22d3	-0.6037846	0.000832998	0.027531492
Prim2	0.85492122	0.000834938	0.027531492
Fam107a	-1.4004723	0.000842687	0.027699379
S100a16	0.50882921	0.000843955	0.027699379
Ripk3	1.19927932	0.000855079	0.027934574
Tpm4	0.88489292	0.000864693	0.028136531
Zfp946	-0.618868	0.000869051	0.028136531
Dixdc1	-0.9034783	0.000869516	0.028136531
Etv4	2.08051458	0.000876911	0.028136531
Diap2	-0.5111788	0.000877211	0.028136531
Amn1	-0.6149765	0.000880371	0.028173746
Sik1	0.82316205	0.000912477	0.028670027
Fam161b	-0.5213468	0.000918221	0.028670027
Ptprs	0.58130489	0.000922734	0.028747338
Cdca7	1.33128661	0.000939247	0.029069268
Chrd	-0.5068741	0.000944736	0.029142742
Fign	-0.9559368	0.00094575	0.029142742
Lgals9	1.02443361	0.00096057	0.029287419
Myo5c	-0.5373314	0.000960822	0.029287419
Cdt1	1.33630732	0.000988856	0.029747633
Tuba1a	0.70025596	0.000994699	0.029775139
Tbxa2r	1.13149991	0.00099581	0.029775139
Myot	-0.7503417	0.001020276	0.030442199
Pcdhb22	0.707139	0.001039825	0.030871075
Tagap	-1.1822508	0.001043049	0.030871075
Tomm5	0.50691484	0.001052016	0.03093145
Myl6	0.60808778	0.001067772	0.031223863
Ctsl	-0.7352928	0.001069421	0.031223863
N4bp2l1	-1.2175271	0.001074833	0.03127654
Ifitm3	0.91992094	0.001097611	0.031808169
Bcl6b	1.03423423	0.001107237	0.032021387
Ccng2	-0.7276263	0.001134518	0.032211199
Tcp11l2	-0.7258191	0.001137105	0.032211199
Tm2d1	-0.5225887	0.001138926	0.032211199
Ntn1	-0.5139756	0.001177104	0.032637453
Ppargc1b	0.71747266	0.001207571	0.033221162
Nos3	0.5051366	0.001218337	0.033337305
Gdf6	1.63754627	0.001226342	0.033476535
Zcwpw1	1.25256307	0.001237092	0.033639845
Itgb3bp	-0.6366654	0.001273882	0.034573641
Prelid1	0.62063779	0.00128234	0.034669847
Sipa1	0.636309	0.001286438	0.034714147

Unc5b	0.83555169	0.001306661	0.035051547
Kdelr2	0.50548007	0.00133172	0.03532812
AA388235	-0.9912709	0.001346318	0.035596336
Rgs16	1.08740281	0.001354643	0.035710936
Otud1	1.50261283	0.001356272	0.035710936
Hirip3	0.61704975	0.001419794	0.036640009
Alpl	0.89680018	0.001420115	0.036640009
F2rl1	1.20725489	0.001424202	0.036655552
Pald1	0.53883454	0.001432762	0.036735532
Cbr2	0.88844917	0.001434229	0.036735532
Rfc5	0.91322547	0.001442406	0.036811347
Cdc45	0.65779576	0.001455468	0.036968265
Abca5	-0.5571019	0.0014567	0.036968265
Sh3d21	-0.6198564	0.001460876	0.036968265
Ckap4	0.57449611	0.00146951	0.036968265
Fam179a	1.15206796	0.001522499	0.037762804
Tfdp2	-0.8347471	0.001532819	0.037865007
Lpcat1	0.51798256	0.001534669	0.037865007
Abcg1	0.91132593	0.001557165	0.038153253
Kcnd3	-0.5951155	0.001585484	0.038579198
Styx	-0.5966648	0.001601781	0.038700334
Bax	0.52478434	0.001604343	0.038700334
Dpm1	-0.5619632	0.001606915	0.038700334
Tigd5	-0.6188753	0.001614289	0.038811686
Oasl1	1.49285204	0.001682177	0.039967288
Atxn10	-0.5330911	0.001694368	0.040189261
Dusp6	0.5944	0.001711547	0.040460733
Ezh1	-0.6533806	0.001725467	0.040653606
Sh3rf2	-0.6124671	0.001744155	0.040957163
Pik3r1	-0.8883435	0.001750146	0.041029586
Pnmt	-1.7296382	0.001776038	0.041361765
Aldob	-1.4229854	0.001785343	0.041448854
Hey2	-0.7366952	0.001791526	0.041448854
Zfp808	-1.2082406	0.001814057	0.041781405
9-Sep	0.63951834	0.001817741	0.041781405
Gpr160	-0.6707277	0.001831366	0.04189643
Tspyl4	-0.6557353	0.00183462	0.04189643
Nrarp	0.9080253	0.001859193	0.04232062
Fkbp5	-1.4059334	0.001871129	0.042425441
Tfec	2.12809826	0.00187824	0.042425441
Plk2	0.58146739	0.001950596	0.043696436
Syne4	-2.0506576	0.001974383	0.044142822
Egr2	1.57068583	0.002000964	0.044471823

Igfbp6	-0.5547504	0.002009805	0.044598073
Sema3f	0.89929273	0.002034684	0.044867955
Gfra2	1.20388639	0.002073649	0.045513857
Prnd	2.24915006	0.002106451	0.045877063
Gm13889	0.78528518	0.002197555	0.047062362
Chtf18	0.96582428	0.002216788	0.047265516
Egr3	1.63439715	0.002217089	0.047265516
Relb	0.51757034	0.002226989	0.047404965
Akap12	0.52887388	0.002241809	0.047515748
Penk	-1.2003109	0.002248279	0.047515748
Fbxl22	0.62350006	0.002251204	0.047515748
S1pr3	0.61821995	0.002253389	0.047515748
Dusp26	-2.0321469	0.002280412	0.047679188
1810041L15Rik	1.42407916	0.002294279	0.047777896
Prim1	1.12225102	0.002321735	0.048115496
Eef2k	-0.5323721	0.002327307	0.048142329
Creb3l1	0.81140803	0.002336963	0.048142329
Gas2l3	1.01182337	0.002337202	0.048142329
Pkmyt1	0.56389256	0.002349746	0.048200533
Csdc2	-0.5595805	0.002368535	0.048515431
Fam150b	-1.5419283	0.002409123	0.049061981
Rpp40	-0.7429875	0.002503389	0.049901589

Supplementary Table 2. DEGs of TAC mice hearts between SD and LCD-P

gene	log2FoldChange	pvalue	adjusted pvalue
Tpst2	0.52694576	1.54E-11	1.69E-07
Lamp2	-0.5250519	7.11E-10	3.90E-06
Tecrl	-1.0164891	1.23E-09	4.50E-06
Smpd1	0.63857205	1.23E-08	3.38E-05
Tmem126a	-0.5427772	2.45E-08	4.48E-05
Tbc1d19	-0.6679687	2.15E-08	4.48E-05
Dbi	0.54592438	5.62E-08	8.81E-05
Ttc14	-0.5596262	1.09E-07	0.000132967
Zfp260	-0.6256912	1.26E-07	0.000137943
Kifc3	0.72503347	2.32E-07	0.00021195
Pnpt1	-0.5020201	3.63E-07	0.000307008
Ppm1k	-0.5982409	4.76E-07	0.000348156
Pgap1	-0.9814339	4.52E-07	0.000348156
Sema7a	0.60322389	8.56E-07	0.000540373
Xpo1	-0.9324618	8.86E-07	0.000540373
Cxadr	-0.5650396	8.42E-07	0.000540373
Tesc	0.74157477	1.30E-06	0.000682292
Eps8	0.60975449	1.95E-06	0.000961063
Abat	-0.6923644	3.02E-06	0.001226356
Scn5a	0.57929437	3.21E-06	0.001257547
Akt1	0.53202771	3.62E-06	0.001363309
Grasp	0.60337721	3.95E-06	0.001400113
Slc2a4rg-ps	-0.7307457	4.64E-06	0.001456447
Zbtb22	0.53256894	4.55E-06	0.001456447
Mroh1	0.57832806	5.06E-06	0.001499088
Zfp397	-0.5076038	5.32E-06	0.001499088
B230354K17Rik	-0.7260838	5.21E-06	0.001499088
Zfp950	-0.6530315	5.63E-06	0.001545917
Pth1r	0.69932334	7.36E-06	0.001759459
Naa30	-0.6653677	7.52E-06	0.001759459
Kctd21	-0.9564388	7.17E-06	0.001759459
Uty	-0.8502323	8.53E-06	0.001950593
Hk1	0.54860343	8.93E-06	0.001982872
Zfp943	-0.8985405	9.62E-06	0.002032663
Plekhm2	0.55361598	1.16E-05	0.002284573
Mir22hg	0.98756254	1.47E-05	0.00265211
Slc25a36	-0.7221795	1.90E-05	0.003255125
Chml	-0.55118	2.32E-05	0.003769782
3110057O12Rik	-0.7582583	2.37E-05	0.003769782
Zfp101	-0.986473	2.34E-05	0.003769782

Kbtbd3	-0.8402623	2.46E-05	0.003841163
Ip6k3	1.2225862	2.53E-05	0.003861519
Zfpm1	0.52683505	3.41E-05	0.004737074
Klhl28	-0.5408494	3.63E-05	0.004976507
Acaa2	0.64611784	3.67E-05	0.004976507
Lrp11	-0.7066007	4.11E-05	0.005254136
Pgp	0.52136517	4.36E-05	0.00550484
Klhl31	-0.5492592	4.49E-05	0.005538757
Tatdn2	0.56982859	4.47E-05	0.005538757
2610305D13Rik	-1.177482	4.76E-05	0.005742892
Zmat1	-0.5349194	4.96E-05	0.005794545
Phospho1	0.5474415	6.41E-05	0.006985565
Abi3	0.50067332	6.70E-05	0.00708713
Plin5	0.8529936	6.92E-05	0.007098596
Tpgs1	0.57752034	6.91E-05	0.007098596
Apoo	-0.8171322	7.42E-05	0.007479892
Orc4	-0.5435688	7.65E-05	0.007640957
Mcts1	-0.5875196	9.61E-05	0.008725634
Tcf7l1	0.52705715	9.99E-05	0.008996041
Ikzf2	-0.5776467	0.000110782	0.009732822
Caprin2	-0.5993905	0.000114053	0.009940746
Cyth1	0.50781097	0.000128062	0.011073847
Acadvl	0.52192154	0.000140674	0.011712142
Ech1	0.85443999	0.000141843	0.011712142
Far1	-0.5460971	0.000152342	0.012301622
Decr1	0.55898726	0.000163014	0.012787253
Mpp7	-0.9644691	0.000160214	0.012787253
Slc38a6	-0.6830746	0.000173367	0.013221623
Ndufa3	0.61988882	0.00017523	0.013271591
Hils1	-0.7967555	0.000195865	0.014239122
Xk	-0.7052141	0.000196484	0.014239122
Sh2d3c	0.52617721	0.00023038	0.015739883
Ramp2	0.5221372	0.000230752	0.015739883
Mrps34	0.50721024	0.000230345	0.015739883
Ppargc1b	0.81463104	0.0002381	0.015811075
Dhrs4	0.51954074	0.000245478	0.015811075
Vwa8	0.59763326	0.000238045	0.015811075
Gpd1	0.85859884	0.00028784	0.017368455
Rac2	-1.5409597	0.000290124	0.017410601
Ccdc28a	-0.5228718	0.000300371	0.01783069
4930430F08Rik	-0.7769847	0.00030451	0.017979192
Vsig10l	0.95896513	0.000315544	0.018142959
Ppargc1a	0.67844376	0.000353286	0.019594863

Zfp703	0.51574898	0.000359008	0.019771723
Zfp800	-0.5728239	0.000374108	0.020338861
Sertad1	0.74914178	0.000381449	0.020635803
Rgcc	0.7648255	0.000403493	0.021374797
Cpt1a	0.53326974	0.000419599	0.021736005
Snta1	0.65629982	0.000424732	0.021898648
Cox10	0.60375751	0.000431477	0.022062059
Alg6	-0.582782	0.000448498	0.022593582
Olfr1033	-0.7641886	0.000457361	0.022696207
Igip	-0.6591159	0.000462358	0.022769597
Cluh	0.52704675	0.000479842	0.023170905
Zbtb26	-0.9021721	0.000499279	0.023518161
4930429F24Rik	-0.6489488	0.000509215	0.023796601
Fbp2	0.70030708	0.000648214	0.027960803
Kcnj11	0.6098631	0.00065335	0.028027695
Plin4	0.85849093	0.000668407	0.028341491
Cirbp	0.79431693	0.000696264	0.029184602
Lsp1	0.72480968	0.000725561	0.02995531
Fam57b	1.04632401	0.000765337	0.03090048
Ap1s2	-0.6951365	0.000768795	0.030926418
Grcc10	0.58217946	0.00077478	0.031053428
Naglu	0.61029736	0.000820485	0.031952354
Slc25a25	0.70176878	0.000835969	0.032415377
Ptgs1	0.60790131	0.000846902	0.032415377
Dll4	0.56091229	0.000850085	0.032415377
Tspyl2	0.5729723	0.000889864	0.033239733
Hist2h2be	-0.6181201	0.000896374	0.033369427
Arhgap27	0.54035501	0.000904074	0.033429429
D17H6S53E	0.53212289	0.00091418	0.033689668
Arsk	-0.6011857	0.000927545	0.033729457
Ypel2	-0.7214137	0.000926097	0.033729457
Tmem189	0.52308612	0.000989726	0.035520184
Chpf2	0.62886377	0.001018349	0.036192602
Serp1	-0.547945	0.00105219	0.036917406
Mylip	0.64644007	0.001057632	0.036990164
Dok7	0.64157445	0.001119653	0.037950717
Tns1	0.62033005	0.001218852	0.039601881
Sorcs2	0.68781891	0.001242651	0.039902889
Tmem71	-0.6337263	0.001268135	0.040134449
Zfp874b	-0.6325278	0.001353288	0.041629717
Tmem116	-0.5524448	0.001400788	0.042731823
Zfp942	-0.7895844	0.001414661	0.042755501
Ankrd33b	0.58178771	0.001409995	0.042755501

Klhl2	0.63235917	0.001414861	0.042755501
4732416N19Rik	-0.6904514	0.00150885	0.04452246
Ccdc9	0.51737541	0.001539101	0.044973376
Zfp874a	-0.6446181	0.001605052	0.046143158
Dzip3	-0.7230061	0.001615355	0.046156806
Hdac9	-0.7319397	0.001645749	0.046668145
Hadha	0.50616437	0.001659519	0.04673034
Myrip	0.70664333	0.001731986	0.04778503
Impa2	0.66298998	0.001785134	0.047932368
Ap3s1	-0.6559769	0.001783922	0.047932368
Tns2	0.52718921	0.001763421	0.047932368
Arhgef19	0.5323893	0.001852453	0.048501981
Rin3	0.57500829	0.001918328	0.0496865

Supplementary Table 3. GO analysis data of TAC mice hearts between SD and LCD-A

ID	Description	pvalue	p.adjust	qvalue	Count	geneID
GO:0006260	DNA replication	2.05E-12	7.92E-09	6.60E-09	23	Mcm3/Prim2/Mcm6/Prim1/Trp53/Cdc6/Wdhd1/Tonsl/Cdc45/Chaf1b/Chtf18/Chaf1a/Pola2/Fen1/Pole/Rfc5/Mcm2/Lig1/Rrm1/Mcm5/Gins2/Cdt1/Pola1
GO:0006261	DNA-dependent DNA replication	2.66E-11	5.13E-08	4.28E-08	17	Mcm3/Prim2/Mcm6/Prim1/Cdc6/Wdhd1/Tonsl/Cdc45/Chtf18/Pola2/Pole/Rfc5/Mcm2/Lig1/Mcm5/Cdt1/Pola1
GO:0006270	DNA replication initiation	8.16E-10	1.05E-06	8.75E-07	9	Mcm3/Mcm6/Cdc6/Cdc45/Pola2/Mcm2/Mcm5/Cdt1/Pola1
GO:0000727	double-strand break repair via break-induced replication	6.78E-09	6.54E-06	5.45E-06	6	Mcm3/Mcm6/Cdc45/Mcm2/Mcm5/Gins2
GO:0033260	nuclear DNA replication	5.17E-08	3.60E-05	3.00E-05	8	Mcm3/Mcm6/Cdc45/Mcm2/Lig1/Mcm5/Cdt1/Pola1
GO:1902969	mitotic DNA replication	5.60E-08	3.60E-05	3.00E-05	6	Mcm3/Mcm6/Cdc45/Mcm2/Lig1/Pola1
GO:0044786	cell cycle DNA replication	1.44E-07	7.94E-05	6.62E-05	8	Mcm3/Mcm6/Cdc45/Mcm2/Lig1/Mcm5/Cdt1/Pola1
GO:0071824	protein-DNA complex subunit organization	4.40E-07	0.0002122	0.00017695	15	Mcm3/Ptma/Mcm6/Hey2/Tspyl4/Smarcb1/Trp53/Cdc45/Chaf1b/Chaf1a/Itgb3bp/Mcm2/Mcm5/Asf1b/Cdt1
GO:0065004	protein-DNA complex assembly	1.03E-06	0.000441	0.00036769	13	Mcm3/Mcm6/Hey2/Tspyl4/Trp53/Cdc45/Chaf1b/Chaf1a/Itgb3bp/Mcm2/Mcm5/Asf1b/Cdt1
GO:0009262	deoxyribonucleotide metabolic process	3.66E-06	0.001412	0.00117715	7	Xdh/Dpyd/Tyms/Nudt1/Nt5c3/Rrm1/Dctd

GO:1903131	mononuclear cell differentiation	4.07E-06	0.0014273	0.00118997	21	Cd46/Prdm1/Trp53/Lgals9/Gadd45g/Prelid1/Ctsl/F2rl1/Pik3r1/Ripk3/Egr3/Lgals1/Egr1/Nrarp/Dll4/Csf1/Shb/Dtx1/Cyp26b1/Relb/Bax
GO:0030217	T cell differentiation	4.55E-06	0.0014614	0.00121838	16	Cd46/Prdm1/Trp53/Lgals9/Gadd45g/Prelid1/Ctsl/Ripk3/Egr3/Egr1/Nrarp/Dll4/Shb/Dtx1/Cyp26b1/Relb
GO:0046073	dTMP metabolic process	7.87E-06	0.0023349	0.00194659	4	Dpyd/Tyms/Nt5c3/Dctd
GO:0050678	regulation of epithelial cell proliferation	8.67E-06	0.0023878	0.00199066	18	Pbld1/Nme1/Etv4/Bmp6/Ctsl/Egr3/Sema5a/Uhrf1/Xdh/Nrarp/Egfl7/Thbs1/Dll4/Dysf/Ift122/Bax/Ccnd1/Apln
GO:0044772	mitotic cell cycle phase transition	1.07E-05	0.0026655	0.00222226	18	Trp53/Cdc6/Plk2/Fam107a/Mtbp/Tcf19/Plk3/Rcc1/Abcb1a/Ccng2/Pole/Pidd1/Ccnd1/Rbl2/Nae1/Cdt1/Fbxl22/Cul4b
GO:0030098	lymphocyte differentiation	1.16E-05	0.0026655	0.00222226	19	Cd46/Prdm1/Trp53/Lgals9/Gadd45g/Prelid1/Ctsl/Pik3r1/Ripk3/Egr3/Lgals1/Egr1/Nrarp/Dll4/Shb/Dtx1/Cyp26b1/Relb/Bax
GO:0007093	mitotic cell cycle checkpoint	1.23E-05	0.0026655	0.00222226	10	Trp53/Cdc6/Plk2/Mtbp/Bcl2l1/Plk3/Pidd1/Ccnd1/Nae1/Cdt1
GO:0072527	pyrimidine-containing compound metabolic process	1.24E-05	0.0026655	0.00222226	7	Nme1/Tk1/Aldh6a1/Dpyd/Tyms/Nt5c3/Dctd
GO:0000082	G1/S transition of mitotic cell cycle	1.37E-05	0.0027868	0.00232332	12	Trp53/Plk2/Fam107a/Mtbp/Tcf19/Plk3/Rcc1/Pole/Pidd1/Ccnd1/Rbl2/Cul4b
GO:0150105	NA	1.92E-05	0.0036964	0.00308166	5	Actg1/Abcb1a/Actb/Actn4/Flna
GO:0050673	epithelial cell proliferation	2.20E-05	0.0039089	0.00325886	19	Pbld1/Nme1/Etv4/Bmp6/Ctsl/Egr3/Sema5a/Tcf19/Uhrf1/Xdh/Nrarp/Egfl7/Thbs1/Dll4/Dysf/Ift122/Bax/Ccnd1/Apln

GO:0009394	2'-deoxyribonucleotide metabolic process	2.33E-05	0.0039089	0.00325886	6	Xdh/Dpyd/Tyms/Nudt1/Nt5c3/Dctd
GO:0019692	deoxyribose phosphate metabolic process	2.33E-05	0.0039089	0.00325886	6	Xdh/Dpyd/Tyms/Nudt1/Nt5c3/Dctd
GO:0006281	DNA repair	2.64E-05	0.004226	0.00352317	20	Mcm3/Mcm6/Trp53/Wdhd1/Tonsl/Cdc45/Chaf1b/Chaf1a/Uhrf1/Fen1/Pole/Rfc5/Zcwpw1/Nudt1/Mcm2/Lig1/Mcm5/Gins2/Cul4b/Pola1
GO:0001936	regulation of endothelial cell proliferation	2.74E-05	0.004226	0.00352317	10	Bmp6/Egr3/Sema5a/Xdh/Nrarp/Egfl7/Thbs1/Dll4/Dysf/Apln
GO:0009162	deoxyribonucleoside monophosphate metabolic process	2.90E-05	0.004299	0.00358403	5	Xdh/Dpyd/Tyms/Nt5c3/Dctd
GO:0006970	response to osmotic stress	3.10E-05	0.0044342	0.00369681	8	Map7/Trp53/Plk3/Abcb1a/Dysf/Relb/Bax/Tsc22d3
GO:0006271	DNA strand elongation involved in DNA replication	3.59E-05	0.004838	0.00403339	4	Mcm3/Pole/Lig1/Pola1
GO:0044843	cell cycle G1/S phase transition	3.64E-05	0.004838	0.00403339	12	Trp53/Plk2/Fam107a/Mtbp/Tcf19/Plk3/Rcc1/Pole/Pidd1/Ccnd1/Rbl2/Cul4b
GO:0044770	cell cycle phase transition	4.12E-05	0.0053012	0.00441957	18	Trp53/Cdc6/Plk2/Fam107a/Mtbp/Tcf19/Plk3/Rcc1/Abcb1a/Ccng2/Pole/Pidd1/Ccnd1/Rbl2/Nae1/Cdt1/Fbxl22/Cul4b
GO:0009176	pyrimidine deoxyribonucleoside monophosphate metabolic process	4.84E-05	0.005828	0.0048588	4	Dpyd/Tyms/Nt5c3/Dctd
GO:0010917	negative regulation of mitochondrial membrane potential	4.84E-05	0.005828	0.0048588	4	Prelid1/Slc25a27/Bax/Bnip3
GO:0001935	endothelial cell proliferation	6.64E-05	0.0077627	0.00647177	10	Bmp6/Egr3/Sema5a/Xdh/Nrarp/Egfl7/Thbs1/Dll4/Dysf/Apln

GO:0007162	negative regulation of cell adhesion	8.58E-05	0.0097332	0.00811452	14	Lgals9/Bmp6/Pik3r1/Fam107a/Sema5a/Lgals1/Abat/Spry4/Cfl1/Nrarp/Thbs1/Dtx1/Plxnd1/Actn4

Supplementary Table 4. GO analysis data of TAC mice hearts between SD and LCD-P

ID	Description	pvalue	p.adjust	qvalue	Count	geneID
GO:0006635	fatty acid beta-oxidation	5.06E-09	7.13E-06	6.41E-06	8	Dbi/Acadvl/Akt1/Plin5/Acaa2/Cpt1a/Decr1/Hadha
GO:0019395	fatty acid oxidation	6.82E-09	7.13E-06	6.41E-06	9	Dbi/Acadvl/Akt1/Plin5/Acaa2/Cpt1a/Decr1/Hadha/Ppargc1a
GO:0034440	lipid oxidation	1.18E-08	8.24E-06	7.41E-06	9	Dbi/Acadvl/Akt1/Plin5/Acaa2/Cpt1a/Decr1/Hadha/Ppargc1a
GO:0009062	fatty acid catabolic process	4.89E-08	2.56E-05	2.30E-05	8	Dbi/Acadvl/Akt1/Plin5/Acaa2/Cpt1a/Decr1/Hadha
GO:0046320	regulation of fatty acid oxidation	6.57E-08	2.75E-05	2.47E-05	6	Dbi/Acadvl/Akt1/Plin5/Cpt1a/Ppargc1a
GO:0030258	lipid modification	1.12E-07	3.89E-05	3.50E-05	11	Dbi/Acadvl/Akt1/Ip6k3/Plin5/Impa2/Acaa2/Cpt1a/Decr1/Hadha/Ppargc1a
GO:0072329	monocarboxylic acid catabolic process	2.11E-07	6.32E-05	5.68E-05	8	Dbi/Acadvl/Akt1/Plin5/Acaa2/Cpt1a/Decr1/Hadha
GO:0006631	fatty acid metabolic process	3.10E-07	8.10E-05	7.28E-05	13	Dbi/Acadvl/Akt1/Plin5/Acaa2/Cpt1a/Ptgs1/Tmem189/Decr1/Hadha/Ppargc1a/Tecrl/Ech1
GO:0046322	negative regulation of fatty acid oxidation	9.87E-07	0.0002128	0.00019129	4	Dbi/Acadvl/Akt1/Plin5
GO:0044282	small molecule catabolic process	1.02E-06	0.0002128	0.00019129	11	Dbi/Hk1/Acadvl/Akt1/Abat/Plin5/Impa2/Acaa2/Cpt1a/Decr1/Hadha
GO:0046395	carboxylic acid catabolic process	1.42E-06	0.0002701	0.00024282	9	Dbi/Acadvl/Akt1/Abat/Plin5/Acaa2/Cpt1a/Decr1/Hadha
GO:0016054	organic acid catabolic process	1.96E-06	0.0003407	0.00030632	9	Dbi/Acadvl/Akt1/Abat/Plin5/Acaa2/Cpt1a/Decr1/Hadha

GO:0044242	cellular lipid catabolic process	2.56E-06	0.0004113	0.00036978	9	Dbi/Acadvl/Akt1/Plin5/Acaa2/Cpt1a/Decr1/Hadha/Smpd1
GO:0031998	regulation of fatty acid beta-oxidation	5.14E-06	0.000768	0.00069046	4	Dbi/Akt1/Plin5/Cpt1a
GO:0019217	regulation of fatty acid metabolic process	1.32E-05	0.0017264	0.00155216	6	Dbi/Acadvl/Akt1/Plin5/Cpt1a/Ppargc1a
GO:0062014	negative regulation of small molecule metabolic process	1.32E-05	0.0017264	0.00155216	6	Dbi/Acadvl/Akt1/Pgp/Plin5/Ppargc1a
GO:0045922	negative regulation of fatty acid metabolic process	1.40E-05	0.0017271	0.00155284	4	Dbi/Acadvl/Akt1/Plin5
GO:0006006	glucose metabolic process	1.81E-05	0.0019867	0.00178622	8	Hk1/Akt1/Fbp2/Gpd1/Pgp/Cpt1a/Serp1/Kcnj11
GO:0042180	cellular ketone metabolic process	1.81E-05	0.0019867	0.00178622	8	Dbi/Acadvl/Akt1/Dhrs4/Gpd1/Plin5/Cpt1a/Ppargc1a
GO:0046486	glycerolipid metabolic process	2.97E-05	0.0031065	0.00279306	10	Pgap1/Dbi/Gpd1/Pgp/Ip6k3/Plin5/Impa2/Cpt1a/Hadha/Far1
GO:0019318	hexose metabolic process	4.50E-05	0.0044844	0.00403186	8	Hk1/Akt1/Fbp2/Gpd1/Pgp/Cpt1a/Serp1/Kcnj11
GO:0007005	mitochondrion organization	6.11E-05	0.0058072	0.00522122	11	Pnpt1/Cox10/Cluh/Akt1/Cxadr/Acaa2/Ppargc1a/Ndufa3/Tmem126a/Slc25a36/Apoo
GO:0016042	lipid catabolic process	6.88E-05	0.0062557	0.00562447	9	Dbi/Acadvl/Akt1/Plin5/Acaa2/Cpt1a/Decr1/Hadha/Smpd1
GO:0005996	monosaccharide metabolic process	7.27E-05	0.0063303	0.00569156	8	Hk1/Akt1/Fbp2/Gpd1/Pgp/Cpt1a/Serp1/Kcnj11
GO:0010565	regulation of cellular ketone metabolic process	9.62E-05	0.0080496	0.00723736	6	Dbi/Acadvl/Akt1/Plin5/Cpt1a/Ppargc1a
GO:0062012	regulation of small molecule metabolic process	0.000112	0.0090371	0.00812519	9	Dbi/Acadvl/Akt1/Gpd1/Pgp/Plin5/Cpt1a/Ppargc1a/Pth1r

Supplementary Table 5. IPA data of TAC mice hearts between SD and LCD-P

Upstream Regulator	Molecule Type	p-value of overlap	Target molecules in dataset
GW501516	chemical drug	2.23E-06	ACAA2,CPT1A,DECR1,ECH1,GPD1,HADHA,PPARGC1A
PNPLA2	enzyme	3.03E-06	ACAA2,ACADVL,CPT1A,PPARGC1A,PPARGC1B
MAP4K4	kinase	5.90E-06	ACAA2,ACADVL,COX10,DHRS4,FAR1,HADHA,NDUFA3
pirinixic acid	chemical toxicant	1.05E-05	ACAA2,ACADVL,CPT1A,DBI,DECR1,ECH1,FBP2,GPD1,HADHA,PLIN4,PLIN5,PPARGC1A
PLIN5	other	2.01E-05	ACAA2,ACADVL,PPARGC1A,PPARGC1B
gemfibrozil	chemical drug	5.90E-05	ACADVL,CPT1A,DECR1,ECH1,HADHA
PPARA	ligand-dependent nuclear receptor	7.26E-05	ACAA2,ACADVL,CPT1A,DBI,DECR1,ECH1,GPD1,HADHA,PLIN4,PLIN5,PPARGC1A
OMA1	peptidase	1.02E-04	ACADVL,PPARGC1A,PPARGC1B
PPARG	ligand-dependent nuclear receptor	1.04E-04	ACAA2,CPT1A,FAM57B,FBP2,GPD1,HADHA,PLIN4,PLIN5,PPARGC1A,PPARGC1B,ZNF703
KLF15	transcription regulator	1.24E-04	ACADVL,DECR1,HADHA,PPARGC1A
fenofibrate	chemical drug	1.40E-04	ACAA2,ACADVL,CPT1A,DBI,DECR1,ECH1,FBP2,HADHA
FGF21	growth factor	1.48E-04	CPT1A,MYLIP,PPARGC1A,PPARGC1B
bezafibrate	chemical drug	2.47E-04	ACADVL,DBI,ECH1,HADHA,PPARGC1A
DMD	other	2.54E-04	GPD1,IMPA2,IP6K3,LAMP2,PPARGC1A,SNTA1,XK
clofibrate	chemical drug	2.85E-04	ACADVL,CPT1A,DECR1,ECH1,HADHA
mono-(2-ethylhexyl)phthalate	chemical toxicant	4.01E-04	ACADVL,FBP2,GPD1,HADHA,PPARGC1A,PPARGC1B
vaccenic acid	chemical - endogenous mammalian	4.96E-04	PLIN4,PLIN5
DIO3	enzyme	7.68E-04	CIRBP,KCNJ11,SEMA7A
GCG	other	8.28E-04	CPT1A,HK1,PPARGC1A,PPARGC1B

rosiglitazone	chemical drug	8.81E-04	ACAA2,ACADVL,CPT1A,DECR1,ECH1,GPD1,PLIN5,PPARGC1A,PPARGC1B
2,4-dinitrophenol	chemical toxicant	9.19E-04	PPARGC1A,PPARGC1B
meldonium	chemical drug	9.19E-04	CPT1A,HADHA
AP3B1	transporter	1.18E-03	AP3S1,LAMP2
D-thioctic acid	chemical - endogenous mammalian	1.18E-03	CPT1A,PPARGC1A
KCNJ11	ion channel	1.18E-03	ACADVL,PPARGC1A
methapyrilene	chemical drug	1.33E-03	ABAT,CPT1A,DBI,DECR1,SERP1
PPARD	ligand-dependent nuclear receptor	1.72E-03	ACAA2,ACADVL,CPT1A,ECH1,GPD1,PPARGC1B
CPT1C	enzyme	1.79E-03	CPT1A,PPARGC1A
eicosapentenoic acid	chemical drug	1.98E-03	ACADVL,ECH1,IMPA2,KCNJ11
PIK3CB	kinase	2.13E-03	DLL4,HK1
INS	other	2.15E-03	ACADVL,CPT1A,PPARGC1A,PTGS1,SMPD1
gamma-linolenic acid	chemical - endogenous mammalian	2.51E-03	PLIN4,PLIN5
HSPA5	enzyme	2.92E-03	ACADVL,CPT1A,HADHA
guanidinopropionic acid	chemical - endogenous non-mammalian	2.92E-03	NDUFA3,PPARGC1A,PPARGC1B
carbonyl cyanide p-(trifluoromethoxy)phenyl hydrazone	chemical reagent	2.92E-03	PPARGC1A,PPARGC1B
methotrexate	chemical drug	3.02E-03	ACAA2,ACADVL,CPT1A,ECH1,FAR1,HADHA
fluocinolone acetonide	chemical drug	3.09E-03	CXADR,LSP1,RGCC
ENPP2	enzyme	3.36E-03	PPARGC1A,PPARGC1B

PLIN1	other	3.36E-03	ACADVL,HADHA
SERTAD2	transcription regulator	3.36E-03	ECH1,PPARGC1A
plicamycin	chemical drug	3.56E-03	EPS8,GPD1,SEMA7A,TESC
fatty acid	chemical - endogenous mammalian	3.68E-03	AKT1,PLIN4,PLIN5,PPARGC1A
IL15	cytokine	3.87E-03	AKT1,CPT1A,DHRS4,HK1,LSP1,PPARGC1A,PPARGC1B,RAC2
FOXO1	transcription regulator	3.93E-03	COX10,GPD1,IKZF2,LAMP2,PPARGC1A,PPARGC1B,PTGS1,SLC25A25
ESRRA	ligand-dependent nuclear receptor	4.28E-03	ACADVL,HADHA,HK1,PPARGC1A,PPARGC1B
ABCC8	transporter	4.31E-03	ACADVL,PPARGC1A
NO 1886	chemical drug	4.31E-03	ACAA2,HADHA
LEP	growth factor	4.68E-03	ACADVL,CPT1A,DLL4,ECH1,FBP2,PPARGC1A,PPARGC1B,SMPD1
SCD	enzyme	4.70E-03	ACADVL,CPT1A,PPARGC1A
PKD1	ion channel	4.79E-03	ALG6,APOO,HDAC9,KLHL28,TNS1
MED30	transcription regulator	5.38E-03	COX10,PPARGC1A
acetyl-L-carnitine	chemical - endogenous mammalian	5.38E-03	CPT1A,PPARGC1A
MEF2C	transcription regulator	5.50E-03	HDAC9,PPARGC1A,PTH1R,SCN5A
NR4A3	ligand-dependent nuclear receptor	5.67E-03	FBP2,PPARGC1A,PPARGC1B
3-aminoisobutanoate	chemical - endogenous mammalian	5.82E-03	CPT1A
AMG-9810	chemical reagent	5.82E-03	PPARGC1A
E. coli 0111:B4 peptidoglycan	chemical - endogenous non-mammalian	5.82E-03	DBI

GW 6471	chemical reagent	5.82E-03	PPARGC1A
OSTN	other	5.82E-03	PPARGC1A
SCN10A	ion channel	5.82E-03	SCN5A
Tug1	other	5.82E-03	PPARGC1A
nicotinamide-beta-riboside	chemical - endogenous mammalian	5.82E-03	PPARGC1A
stearidonic acid	chemical - endogenous mammalian	5.82E-03	PTGS1
TFAM	transcription regulator	5.96E-03	ECH1,HK1
BSCL2	other	6.46E-03	GPD1,PPARGC1B,PTGS1
PD 169316	chemical - kinase inhibitor	6.56E-03	PPARGC1A,PPARGC1B
HIPK2	kinase	7.03E-03	PPARGC1A,RAC2,ZFPM1
zymosan	chemical - endogenous non-mammalian	7.32E-03	PPARGC1A,PPARGC1B,PTGS1
RB1	transcription regulator	7.79E-03	AKT1,EPS8,HIST1H2BJ,NDUFA3,PPARGC1A,PPARGC1B,PTGS1
mir-33	microrna	7.85E-03	CPT1A,PPARGC1A
zidovudine	chemical drug	7.85E-03	AKT1,CPT1A
triamcinolone acetonide	chemical drug	7.90E-03	AP3S1,CXADR,LSP1,RGCC,TSPYL2
VEGFA	growth factor	7.91E-03	AKT1,DECR1,DLL4,HK1,PPARGC1A,PTGS1
PRNP	other	7.93E-03	EPS8,LAMP2,TCF7L1
linoleic acid	chemical - endogenous mammalian	8.25E-03	CPT1A,PLIN4,PLIN5
mir-17	microrna	8.25E-03	AKT1,CAPRIN2,MYLIP
INSR	kinase	8.44E-03	ACAA2,ACADVL,DECR1,HADHA,NDUFA3,PPARGC1A,PPARGC1B

GW7647	chemical drug	8.53E-03	CPT1A,PPARGC1A
Calcineurin A	group	9.23E-03	AKT1,PPARGC1A
HAND1	transcription regulator	9.23E-03	CPT1A,DBI
TP53	transcription regulator	9.25E-03	ABAT,ACAA2,ACADVL,AKT1,CLUH,COX10,CPT1A,DBI,ECH1,EPS8,GPD1,HADHA,HDAC9,LSP1,PPARGC1A,PPARGC1B,PTGS1,RAC2,XPO1
tretinoin	chemical - endogenous mammalian	9.33E-03	AKT1,AP1S2,C12orf29,CPT1A,DBI,DLL4,GRASP,HADHA,HK1,LAMP2,LSP1,PPARGC1A,PTGS1,SEMA7A,SLC25A36,SMPD1,XK
TGM2	enzyme	1.04E-02	C12orf29,LSP1,PTGS1,SEMA7A,SMPD1
LPIN1	phosphatase	1.07E-02	ACADVL,CPT1A
PPP3CA	phosphatase	1.10E-02	CPT1A,DECR1,LAMP2
MTOR	kinase	1.15E-02	HADHA,PGP,PPARGC1A,PPARGC1B,PTH1R,SMPD1
CIDEC	other	1.15E-02	PPARGC1A,PPARGC1B
MEF2	group	1.15E-02	DLL4,PPARGC1A
stearic acid	chemical - endogenous mammalian	1.15E-02	PPARGC1A,PPARGC1B
KCP	other	1.16E-02	PPARGC1A
MLIP	other	1.16E-02	HK1
Mitochondrial complex 1	complex	1.16E-02	PPARGC1A
RPS6KA1	kinase	1.16E-02	SEMA7A
SLC20A1	transporter	1.16E-02	AKT1
SLC25A33	transporter	1.16E-02	PPARGC1A
canertinib	chemical drug	1.16E-02	AKT1
cyclic des-acyl ghrelin (6-13)	biologic drug	1.16E-02	PPARGC1A

foscarnet	chemical drug	1.16E-02	AKT1
mir-374	microrna	1.16E-02	AKT1
prostaglandin E3	chemical - endogenous mammalian	1.16E-02	PTGS1
ELOVL5	enzyme	1.23E-02	HADHA,PPARGC1A
MLXIPL	transcription regulator	1.31E-02	CPT1A,GPD1
PRKCI	kinase	1.31E-02	HK1,KCNJ11
PPARGC1A	transcription regulator	1.49E-02	ACADVL,CPT1A,PLIN5,PPARGC1A,PPARGC1B
DIO2	enzyme	1.58E-02	CIRBP,SEMA7A
HSD11B1	enzyme	1.58E-02	CPT1A,PPARGC1A
RAB1B	other	1.58E-02	CYTH1,SERP1
SLC16A2	transporter	1.58E-02	CIRBP,SEMA7A
Ank2	other	1.74E-02	KCNJ11
DGCR5	other	1.74E-02	AKT1
DUB	group	1.74E-02	MYLIP
HAO1	enzyme	1.74E-02	PPARGC1A
HCFC1	transcription regulator	1.74E-02	PPARGC1B
SLC30A7	transporter	1.74E-02	AKT1
SLN	other	1.74E-02	PPARGC1A
Trk Receptor	group	1.74E-02	AKT1
Z-LEHD-FMK	chemical reagent	1.74E-02	AKT1
mecamylamine	chemical drug	1.74E-02	KCNJ11
mir-1180	microrna	1.74E-02	AKT1
mir-149	microrna	1.74E-02	AKT1
natriuretic peptide derivative	biologic drug	1.74E-02	PPARGC1A

stavudine	chemical drug	1.74E-02	CPT1A
nafenopin	chemical drug	1.96E-02	CPT1A,DECR1
FOXA3	transcription regulator	2.06E-02	CPT1A,PPARGC1A
tetraethylammonium	chemical drug	2.27E-02	ECH1,HADHA
2-[3-chloro-4-[3-[(3-phenyl-7-propyl-1-benzofuran-6-yl)oxy]propylsulfanyl]phenyl]acetic acid	chemical reagent	2.31E-02	CPT1A
ABCC9	ion channel	2.31E-02	PPARGC1A
ACOT11	enzyme	2.31E-02	PPARGC1A
HCAR1	g-protein coupled receptor	2.31E-02	PPARGC1A
SCIN	other	2.31E-02	RAC2
miR-515-5p (and other miRNAs w/seed UCUCCAA)	mature microrna	2.31E-02	TCF7L1
nebivolol	chemical drug	2.31E-02	PTGS1
pterosin B	chemical reagent	2.31E-02	PPARGC1A
trimetazidine	chemical drug	2.31E-02	PPARGC1A
troglitazone	chemical drug	2.33E-02	ARHGEF19,DBI,ECH1,GPD1,PLIN4,PPARGC1A
CTGF	growth factor	2.35E-02	AKT1,PTH1R,RAMP2
PRKAA	group	2.38E-02	CPT1A,PPARGC1A
NOS2	enzyme	2.43E-02	GPD1,PPARGC1A,PTGS1,TSPYL2
nicotine	chemical drug	2.43E-02	AKT1,DBI,KCNJ11,PTH1R
Esrra	transcription regulator	2.46E-02	CPT1A,ECH1,PPARGC1A

8-epi-prostaglandin F2alpha	chemical - endogenous mammalian	2.88E-02	PTH1R
ACOT13	enzyme	2.88E-02	PPARGC1A
FOXN4	transcription regulator	2.88E-02	DLL4
LAMP1	other	2.88E-02	LAMP2
MYBBP1A	transcription regulator	2.88E-02	PPARGC1A
Nfatc	group	2.88E-02	PPARGC1A
SLC22A2	transporter	2.88E-02	CPT1A
SOX18	transcription regulator	2.88E-02	DLL4
ZFA-fmk	chemical toxicant	2.88E-02	LAMP2
aurapten	chemical - endogenous non-mammalian	2.88E-02	CPT1A
globotriaosylceramide	chemical - endogenous mammalian	2.88E-02	LAMP2
mir-637	microrna	2.88E-02	AKT1
oleic acid	chemical - endogenous mammalian	2.90E-02	PLIN4,PLIN5,PPARGC1A
cisplatin	chemical drug	2.92E-02	ACADVL,AKT1,CPT1A,HADHA,HDAC9,HK1,PPARGC1A,SLC25A36,SMPD1
TGFB1	growth factor	2.98E-02	ACAA2,AKT1,AP3S1,ARHGEF19,CCDC28A,CXADR,DZIP3,HDAC9,PPARGC1A,PTGS1,PTH1R,RAMP2,RGCC,SEMA7A,SERP1,SERTAD1,TPST2
GLI1	transcription regulator	3.10E-02	ACADVL,AKT1,IMPA2,PTH1R
NR1H3	ligand-dependent nuclear receptor	3.23E-02	HK1,MYLIP,PPARGC1A
ERBB2	kinase	3.24E-02	ACAA2,AKT1,DECR1,DZIP3,KIFC3,LAMP2,PTGS1,SERP1,ZNF703

D-glucose	chemical - endogenous mammalian	3.36E-02	ACAA2,DBI,DECR1,FBP2,GPD1,HK1,PPARGC1A,XK
EPB41	other	3.44E-02	SCN5A
ERF	transcription regulator	3.44E-02	SEMA7A
HMG20A	transcription regulator	3.44E-02	CXADR
KRIT1	other	3.44E-02	DLL4
KX-01	chemical drug	3.44E-02	AKT1
OBP2B	transporter	3.44E-02	CPT1A
PDZK1IP1	other	3.44E-02	PTGS1
PITPNA	transporter	3.44E-02	PTGS1
SPTBN4	other	3.44E-02	KCNJ11
XPA	other	3.44E-02	PTGS1
taspoglutide	biologic drug	3.44E-02	CPT1A
CEBPB	transcription regulator	3.56E-02	CIRBP,CPT1A,PPARGC1A,PTGS1,RAC2,SERP1
BMP7	growth factor	3.59E-02	AKT1,PPARGC1A,PPARGC1B
FST	other	3.69E-02	PPARGC1A,PPARGC1B
MSTN	growth factor	3.69E-02	AKT1,PPARGC1A
ZBTB20	transcription regulator	3.69E-02	AKT1,PPARGC1A
HDAC4	transcription regulator	3.74E-02	DLL4,HDAC9,KLHL2
FNIP1	other	4.00E-02	PPARGC1A
ITGB1BP1	transporter	4.00E-02	DLL4
MARCKSL1	other	4.00E-02	SCN5A
PQQ cofactor	chemical - endogenous non-mammalian	4.00E-02	PPARGC1A
RIOX1	enzyme	4.00E-02	AKT1
SLC22A1	transporter	4.00E-02	CPT1A

ZNF746	transcription regulator	4.00E-02	PPARGC1A
branched chain amino acids	chemical drug	4.00E-02	PPM1K
mir-634	microrna	4.00E-02	LAMP2
PIM1	kinase	4.08E-02	AKT1,PPARGC1A
STAT4	transcription regulator	4.21E-02	PGP,RAMP2,RGCC,SERTAD1
SIRT6	enzyme	4.22E-02	AKT1,PPARGC1A
telmisartan	chemical drug	4.22E-02	CPT1A,PTH1R
TCF7L2	transcription regulator	4.49E-02	FBP2,KLHL2,PPARGC1A,TNS1,YPEL2
3,5-L-diiodothyronine	chemical - endogenous mammalian	4.56E-02	CPT1A
CAMKK2	kinase	4.56E-02	PPARGC1A
KDM6A	enzyme	4.56E-02	Uty
KSR1	kinase	4.56E-02	PPARGC1B
OCT4-NANOG	complex	4.56E-02	TCF7L1
TRIB1	kinase	4.56E-02	CPT1A
cosyntropin	biologic drug	4.56E-02	DLL4
miR-217-5p (and other miRNAs w/seed ACUGCAU)	mature microrna	4.56E-02	PPARGC1A
naproxen	chemical drug	4.56E-02	PTGS1
oleoylethanolamide	chemical - endogenous mammalian	4.56E-02	CPT1A
n-nitrosomethylbenzylamine	chemical toxicant	4.60E-02	AP3S1,LAMP2,PTGS1

quercetin	chemical drug	4.60E-02	ACAA2,ECH1,PPARGC1A
Calmodulin	group	4.63E-02	PHOSPHO1,YPEL2
calcitriol	chemical drug	4.84E-02	AKT1,CPT1A,ECH1,PNPT1,RGCC,SLC25A36,XPO1
IKZF1	transcription regulator	4.85E-02	4732416N19Rik,PTH1R,ZFPM1

Supplementary Table 6. Sequences of the primers used for RT-qPCR

Species	Gene	Forward (5'–3')	Reverse (5'–3')
Mouse	*18S*	CTTAGAGGGACAAGTGGCG	ACGCTGAGCCAGTCAGTGTA
Mouse	*Nppb*	AGGGAGAACACGGCATCATT	GACAGCACCTTCAGGAGAT
Mouse	*Cpt1b*	CCCATGTGCTCCTACCAGAT	CCTTGAAGAAGCGACCTTTG
Mouse	*Il6*	TAGTCCTTCCTACCCCAATTTCC	TTGGTCCTTAGCCACTCCTTC
Mouse	*Lcad*	GTAGCTTATGAATGTGTGCAACTC	GTCTTGCGATCAGCTCTTTCATTA
Mouse	*Mcad*	GATCGCAATGGGTGCTTTTGATAGAA	AGCTGATTGGCAATGTCTCCAGCAAA
Mouse	*Plin5*	TGTCCAGTGCTTACAACTCGG	CAGGGCACAGGTAGTCACAC
Mouse	*Ppara*	AGAGCCCCATCTGTCCTCTC	ACTGGTAGTCTGCAAAACCAAA
Mouse	*Tnf*	CAGGCGGTGCCTATGTCTC	CGATCACCCCGAAGTTCAGTAG
Rat	*Acaa2*	CAGAGGTGGAAAGCCGCTAA	TGCTCATCCACTTGCATGGT
Rat	*Nppa*	ATCTGATGGATTTCAAGAACC	CTCTGAGACGGGTTGACTTC
Rat	*Nppb*	GCTTTGGGCAGAAGATAGACC	AGAGCTGGGGAAAGAAGAGC
Rat	*Atgl1*	CCTGACTCGAGTTTCGGAT	CACATAGCGCACCCCTTGA
Rat	*Cpt1a*	CAGCTCGCACATTACAAGGA	TGCACAAAGTTGCAGGACTC
Rat	*Lcad*	AACCAAACGTCTGGACTCCG	GCATCCACGTAGGCTTTTGC
Rat	*Plin5*	GGATGTCCGGTGATCAGAC	GTGCACGTGGCCCTGACCAG
Rat	*Ppara*	TCGTGGAGTCCTGGAACTGA	CTTCAGTCTTGGCTCGCCTC

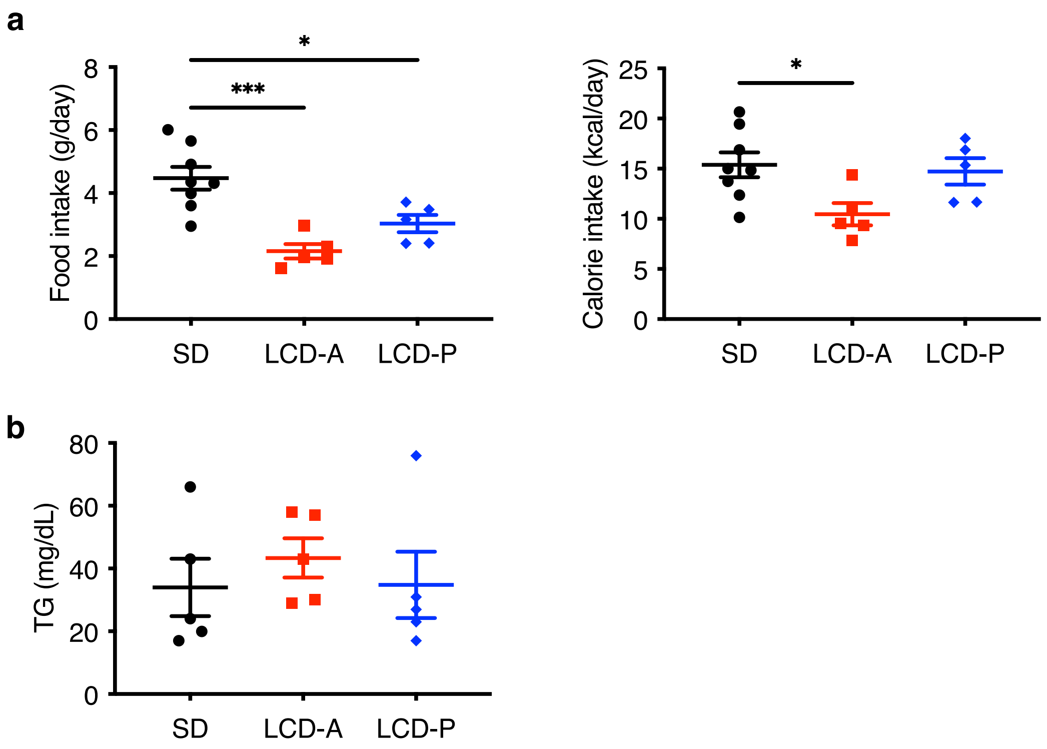

Supplementary Figure 1. Food intake and serum triglyceride levels among diet groups.
a Food intake and calorie intake of mice fed the indicated three diets (n = 5-8). **b** Serum triglyceride (TG) levels of mice fed the indicated three diets for 4 weeks (n = 5). Data are shown as mean ± SEM. One-way ANOVA followed by Holm-Sidak's post-hoc test. *$P < 0.05$, **$P < 0.01$, ***$P < 0.001$.
